# Morphine Equianalgesic Dose Chart in the Emergency Department

**DOI:** 10.21980/J8RD29

**Published:** 2022-07-15

**Authors:** Savannah Tan, Ellen Lee, Stephen Lee, Sangeeta S Sakaria, Jennifer S Roh

**Affiliations:** * University of California Irvine, Department of Emergency Medicine, Orange, CA

## Abstract

**Audience:**

The lecture and infographic are targeted towards Emergency Medicine physicians and residents.

**Introduction:**

Pain is the most common presenting symptom in the emergency department.^1^ Various classes of medications are used to treat acute and chronic pain. Specifically, opiate medications are often used to relieve moderate to severe pain. About 20% of patients presenting with the chief complaint of non-cancer pain receive an opioid prescription.^2^ Since there are many different types of opiates, conversion between one opioid to another has provided a great challenge in terms of addressing the balance between adequately controlling patients’ pain and preventing serious adverse effects. The lack of a readily available standard opiate equivalent guide and physicians’ limited knowledge base about morphine milligram equivalents may contribute to medication errors, insufficient treatment, addiction, and overdose.

**Educational Objectives:**

The primary aim of this study was to educate residents and attending physicians about opiate equivalent medications, medication metabolism, provide usual dosages, and to provide a standardized method for converting between various opiate medications in the emergency department (ED). By the end of this session, the learner will be able to: 1) define the term, “morphine milligram equivalents;” 2) describe the relative onset and duration of action of different pain medications often used in the emergency department; and 3) convert one opioid dose to another.

Additionally, we aimed to evaluate the efficacy of the lecture and the infographic in increasing physicians’ knowledge base of opioid medications and standardize the method of prescribing and converting between opioids in the ED. We designed and placed a simple, eye-catching infographic in the University of California, Irvine Emergency Department that depicted information pertaining to morphine equivalents and pharmacological properties of the opioids. We then presented a lecture on morphine equianalgesic doses, metabolism, and method for conversion between medications. In order to evaluate the functionality of the lecture and chart, we administered multiple surveys to ED providers pre- and post-lecture and placement of the chart in the ED. Our lecture and infographic included up-to-date literature and considered dose reductions, cross tolerance, and patient comorbidities. We designed the infographic to be visually appealing and simple for ease of use in a busy ED environment.

**Educational Methods:**

A lecture was designed to educate emergency department physicians and residents on the properties, metabolism and techniques for conversion between various opioid medications. Following the lecture, we walked through an example question with the participants. The lecture was presented at an Emergency Department conference.

**Research Methods:**

This lecture was presented at emergency medicine residency grand rounds. To evaluate the efficacy of our chart and educational lecture, we implemented a pre-presentation survey consisting of questions related to opiate conversions, metabolism, and medication characteristics without the help of the chart. After the presentation participants were once again asked to fill out the same set of questions on a post-presentation survey with the help of the chart. The effectiveness of the lecture and infographic was assessed by comparing participants’ scores between the pre-presentation survey and post-presentation survey. A second post-presentation survey with the same set of questions was also sent out about 7 months after the presentation, to assess for retention of the information presented during the lecture. The responses were kept anonymous, though participants were asked for their level of training and four screening questions for the purpose of matching individual responses between the pre-presentation, initial post-presentation, and 7-month post-presentation surveys.

**Results:**

Seven initial post-presentation survey responses were matched to pre-presentation survey responses, while five 7-month post-presentation survey responses were matched to pre-presentation survey responses. Only one participant filled out all three surveys; this participant was found to have increased in score from pre-presentation survey (4/10) to immediate post-presentation survey (10/10), but decreased in score at the 7-month post-presentation survey (8/10). Five of the participants who filled out both the pre- presentation survey and immediate post-presentation survey showed improvement in their scores, one participant received the same score on both surveys, and one participant had a decrease in score. Between the five participants who filled out the pre-presentation survey and the 7-month post-presentation survey, one participant showed an increase in scores, two participants received the same score each time, and two participants decreased in scores.

**Discussion:**

Overall, the educational content and infographic allowed for improvement in a majority of the participants’ scores between the pre-presentation survey and immediate post-presentation survey. However, it seems that retention of knowledge gained by the presentation waned as time passed, which manifested in most participants showing a decrease or no change in score between the pre-presentation survey and the 7-month post-presentation survey. In other words, through implementation of the infographic in the Emergency Department and educational lecture, we learned that the presentation and the walk-through of an example question contributed to immediate retention of knowledge of morphine equivalents. However, long-term retention of the knowledge about morphine equivalents was lacking. Given the small sample size of eleven participants, we are unable to definitively conclude whether this infographic and lecture are overall effective in improving knowledge, retention of knowledge, and change in clinical management. However, our results suggest that further larger studies could be conducted with the infographic and presentation as useful tools to advance EM physicians’ knowledge and awareness of morphine milligram equivalents. Therefore, our hypothesis still stands that this infographic and lecture are useful tools that other EM programs could use and conduct studies to evaluate improvement in their learners’ knowledge. The main takeaways are that educational lectures and visually-appealing graphics are able to enhance physicians' understanding of morphine equianalgesic doses in the immediate period after exposure, but must also be conducted with consistent follow-up to improve preservation of knowledge.

**Topics:**

Morphine milligram equivalents, morphine equianalgesic doses, opioids, opiates, infographic.

## User Guide

**Table:** 

**List of Resources:**	
Abstract	1
User Guide	4
Appendix 1: Morphine Equivalents Lecture PowerPoint	6
Appendix 2: Morphine Equivalents Lecture Script	7
Appendix 3: Opioid Equianalgesic Chart	15
Appendix 4: CDC Calculating Total Daily Dose Of Opioids For Safer Dosage	16
Appendix 5: Morphine Equivalents Survey Questions	17
Appendix 6: Morphine Equivalents Survey Answers	19

**Learner Audience:**

Interns, Junior Residents, Senior Residents, Attending Physicians

**Time Required for Implementation:** 20 minutes

**Recommended Number of Learners per Instructor:**

There is no maximum or minimum number of learners who can participate in this lecture.

**Topics:**

Morphine milligram equivalents, equianalgesic dosage, opioid.

**Objectives:**

By the end of this session, the learner will be able to:

1. Define the term, “morphine milligram equivalents”
2. Describe the relative onset and duration of action of different pain medications often used in the emergency department
3. Convert one opioid dose to another.

### Linked objectives and methods

The lecture provides an in-depth description of morphine milligram equivalents and the specific characteristics of common pain medications used in the emergency department. We also included a case example that applies the concepts taught during the lecture to walk the learners through how to convert between oxycodone and morphine to solidify the main objective of the lecture. Because there is an overall lack of opiate equivalent guides and visual aids for quick reference when converting between two different pain medications, we designed an educational lecture and a visually appealing chart aimed towards teaching Emergency Medicine residents and attending physicians the importance of opiate equivalents and opioid metabolism. The infographic was created with the goal of developing an attractive but simple chart that would aid learners in easily converting between the medications. Our primary goal in developing this curriculum was to encourage familiarity with the concept of morphine milligram equivalents such that learners would feel more comfortable prescribing various opioids and converting between them.

### Recommended pre-reading for instructor

1. Todd KH, Ducharme J, Choiniere M, et al. PEMI Study Group. Pain in the emergency department: results of the pain and emergency medicine initiative (PEMI) multicenter study. *J Pain*. 2007 Jun;8(6):460–6. Epub 2007 Feb 15. PMID: 17306626. doi:10.1016/j.jpain.2006.12.005
2. CDC Calculating Total Daily Dose of Opioids for Safer Dosage. At: https://www.cdc.gov/drugoverdose/pdf/calculating_total_daily_dose-a.pdf
3. Maryland Department of Health Morphine Milligram Equivalents Fact Sheet. At: https://health.maryland.gov/pdmp/Documents/MME%20Fact%20Sheet_2.24.2020.pdf

### Results and Tips for Successful Implementation

The lecture is best implemented in a setting when learners are able to solely focus on the topic without distractions and in a collaborative environment that encourages participation in and discussion during the example question. Implementation was tested during the University of California, Irvine (UCI) Emergency Medicine residency conference with a pre- presentation survey, immediate post-presentation survey, and a 7-month follow-up post-presentation survey. First-, second-, and third-year residents were present at the conference. Participants were asked to take the pre-presentation survey at their baseline knowledge without any context of morphine milligram equivalents. The lecture was then given during the conference, which covered key concepts pertaining to the topic of morphine equianalgesic doses and the survey questions, with encouragement to participate in the case example. At the end of the lecture, learners were asked to fill out the immediate post-presentation survey, which consisted of the same questions as the pre-presentation survey. This time, they were able to use the visual aid for reference. The infographic was also placed in the UCI Emergency Department for reference during clinical practice. Finally, a third survey with the same questions was sent out to both residents and attendings, to evaluate long-term retention of the learning materials. Responses were kept anonymous but the surveys included identical screening questions in an attempt to match participants across the three sequential surveys.

Overall, we received 29 responses to the pre-survey, 11 responses to the immediate post-presentation survey, and 11 responses to the 7-month follow-up post-presentation survey. After matching responses by screening questions, we received a total of seven responses for the immediate post-presentation survey that were able to be reliably paired to pre-presentation responses, and five responses for the 7-month follow-up post-presentation survey that were able to be paired to pre-presentation responses. Of those pairs, only one participant was found to have responded to all three surveys longitudinally; this learner exhibited an increase in score from the pre-presentations survey of 40% to 100% in the immediate post-presentation survey but experienced a decrease in score to 80% in the 7-month follow-up survey. Of the seven individuals who filled out both the pre-presentation survey and immediate post-presentation survey, five participants showed improvement in their scores with an average of 38% on the pre-presentation survey and an increase to a mean of 82% on the immediate post-presentation survey; one participant received the same score of 20% on both surveys; and one participant exhibited a decrease in score from 50% to 20%. Of the five individuals who filled out both the pre-presentation survey and the follow-up post-presentation survey, one participant showed an increase in score from 40% to 80%, two participants showed no change in scores with both scoring 60% on the surveys, and two participants had a decrease in scores with an average score of 75% on the pre-presentation survey and a mean score of 65% on the 7-month follow-up post-presentation survey.

The results of the learner who participated in all three surveys suggests that the lecture and infographic were successful in boosting participants’ knowledge in the short-term; it also indicates that our references improved the participants’ knowledge base in the long term, exhibited by the increase in the 7-month follow-up survey score as compared to the pre-presentation survey score. Similarly, the overall increase in the average scores from the pre-presentation survey to immediate post-presentation survey exhibits the effectiveness of the lecture in achieving our defined objectives. However, the slight decrease in retained knowledge on follow-up surveys suggests that closer follow-up and increased instances of education on the topic of morphine milligram equivalents should also be implemented to encourage, improve, and sustain long-term retention of the topic.

### Associated content (optional)

- Appendix 1: Morphine Equivalents Lecture PowerPoint
- Appendix 2: Morphine Equivalents Lecture Script
- Appendix 3: Opioid Equianalgesic Chart
- Appendix 4: CDC Calculating Total Daily Dose Of Opioids For Safer Dosage
- Appendix 5: Morphine Equivalents Survey Questions
- Appendix 6: Morphine Equivalents Survey Answers

### Additional Resources

1. Pain in the emergency department: results of the pain and emergency medicine initiative (PEMI) multicenter study: https://pubmed.ncbi.nlm.nih.gov/17306626/
2. Ambulatory diagnosis and treatment of nonmalignant pain in the United States, 2000–2010: https://pubmed.ncbi.nlm.nih.gov/24025657/
3. Maryland Department of Health Morphine Milligram Equivalents Fact Sheet: https://health.maryland.gov/pdmp/Documents/MME%20Fact%20Sheet_2.24.2020.pdf
4. Link to the Google Drive with the Morphine Milligram Equivalents Lecture Video: https://drive.google.com/drive/folders/1OIJ5GKhp8zo-0VG--WCLLGrYFkG2-ain?usp=sharing

### Technology necessary

A computer or cellular device is necessary to complete the course surveys.

### Assessment (optional)

Participants should be asked to fill out the survey before receiving the lecture without the help of the infographic, and then asked to fill out the same set of questions after the presentation and with the visual aid accessible. A follow-up post-presentation survey may also be implemented after an adequate time to evaluate for retention of the information presented during the lecture. (survey attached)

## USER GUIDE AND LEARNER MATERIALS

### Appendix 1 Morphine Equivalents Lecture PowerPoint

Please see associated PowerPoint file

### Appendix 2 Morphine Equivalents Lecture Script

#### Slide 1

The objective of this lecture is to present the topic of morphine milligram equivalents, to briefly go over the characteristics and metabolism of opioid medications, and to introduce a visually appealing chart that will help guide Emergency Department (ED) providers in roughly converting between commonly used opioids in the ED, specifically in this crucial time period of the opioid crisis.

#### Slide 2

This slide introduces some of the risk factors for opioid overdose/dependence. This is far from a comprehensive list, but just aims to highlight some of the more common etiologies that lead to opioid overuse.

For example, the desire for analgesia (pain relief) may be so strong that it blinds the user from following the recommended dosages and frequency of use.

Lower socioeconomic status also increases the likelihood of opioid overdose, as a result of lower education attainment, employment status, and income level, among other factors.

In regards to euphoria - opioids used for pain relief have analgesic and depressive central nervous system effects and have the potential to cause euphoria. Many people can easily become addicted to this feeling of exhilaration, quickly increasing their usage of opioid medication to chase the “high,” and building tolerance to these medications, thus requiring increasing dosages to reach the same level of euphoria.

Examples of psychiatric comorbidities that increase the risk of developing opioid dependence include depression, anxiety disorder, personality disorder, PTSD, and substance use disorder, among others.

#### Slide 3

Long-term opioid use often begins with treatment of acute pain. Clinicians should prescribe the lowest effective dose of immediate-release opioids and should prescribe no greater quantity than needed for the expected duration of pain severe enough to require opioids.

According to the CDC, greater than 100 million Americans have some source of chronic pain that affects their well-being, level of functioning, and quality of life. They often end up turning to and becoming dependent on pain medication as their primary source of relief. This leads to over 100 Americans dying each day as a result of this dependency and subsequent opioid overdose. In the year 2018 alone, approximately 70% of deaths caused by drug overdose in the United States involved opioid usage.

#### Slide 4

The objective of this educational lecture is to explain how to correctly and safely convert between various pain medications and standardize a method to do so. We will be focusing specifically on the more commonly used drugs in the emergency department.

#### Slide 5

First, we have to define the term “morphine milligram equivalents.” This term refers to the amount of morphine that an opioid dose is equal to when prescribed. The number of morphine milligram equivalents is often used as a reference or standardized gauge of a medication’s potential to be abused as a mechanism of overdose. Specifically, it is possible to “add up” the total morphine milligram equivalents a patient may be taking, even though they are using multiple different opioids at different dosages. For example, if a patient is receiving both Dilaudid (or hydromorphone) and oxycodone during their visit to the emergency department, both Dilaudid and oxycodone can be converted into their respective morphine milligram equivalents, then added up to give you a total.

But not only is the chart and the idea of morphine milligram equivalents useful for calculating a sum of the pain medications a patient is using, you can also use the chart as a guide to switch a patient to an equivalent dose of a different pain medication. For instance, if a patient is receiving a certain dose of IV morphine, but you wish to transition the patient to an oral medication to go home with, you can easily use the morphine equivalents guide to convert that dose to an oxycodone tablet.

#### Slide 6

This is a picture of the actual infographic that will be placed in various spots throughout the UCI emergency department physician room for reference. As you can see, the chart is split up vertically, with the pain medications that are offered as IV on the left and in blue, and the PO pain medications on the right in orange. The medications are ordered from top to bottom by the strongest to the weakest pain medications. There are also icons of both a kidney and liver that represent medications that are cautioned against in patients with renal or hepatic failure or impairment.

In this case, morphine milligram equivalents are determined with the dosage of 30 milligrams of PO morphine as the baseline. In other words, 30 mg of PO morphine is the reference to which other pain medications are compared. Under each opioid, the chart lists the dosage of each medication that is equivalent to 30 milligrams of PO morphine. For example, 0.1 mg of IV fentanyl is equal to 30 milligrams of PO morphine, and so on. The chart also details the onset and duration of action for each pain medication. Please note that there is a disclaimer at the bottom left of the chart that states, when converting from one opioid to another, decrease the dose by 25–50% to account for cross-tolerance, specifically in patients who have had long-term use of opioids.

#### Slide 7

This is a more simple chart to more clearly explain the same idea of morphine equivalents. At the top, you can see that 30 mg of PO morphine (immediate release) is equal to 10 mg of IV morphine. In the same way, you can extrapolate the chart and understand that 30 mg of PO morphine is equal to 30 mg of PO hydrocodone, and 7.5 mg of PO hydromorphone (Dilaudid) is equal to 20 mg of PO oxycodone.

#### Slide 8

Earlier in the lecture, we discussed the concept of morphine milligram equivalents (MME). Hydrocodone and oxycodone with acetaminophen are common oral opioids used in the ED. This table here provides a quick reference for how much morphine equivalents per day a single dose of a 5–325mg tablet (a common x1 dose) would equal.

#### Slide 9

Morphine acts as our baseline standard for all opioid ratio-conversions. For example, 10mg of IV morphine serves as the typical equivalent dose to other parenteral opioid options such as hydromorphone or fentanyl, as shown in the chart earlier. It is commonly given orally or intravenously, although other routes can be used. With the IV form, the onset of action is generally around 5–10 minutes and lasts for 3–4 hours.

#### Slide 10

With oral morphine, the onset of action is around 30 minutes with a duration of 3–6 hours. The typical equianalgesic conversion dose is 30 mg.

One of the key things to remember about morphine in regards to patient safety is that it should typically be avoided in patients with renal failure (not necessarily a contraindication though). Morphine is hepatically metabolized into two main active metabolites, morphine-3-glucuronide (M3G) and morphine-6-glucuronide (M6G) which are excreted in the urine. These metabolites can still exert a physiologic effect and even be more potent than the parent compound. Thus, with renal failure, these metabolites can accumulate in the body causing an increased sensitivity to morphine and increasing the risk of adverse effects such as respiratory depression.

#### Slide 11

Hydrocodone is a commonly used oral opioid (typically in combination with acetaminophen). Its onset of action and duration is similar to oral morphine. The rough conversion between oral morphine and hydrocodone is about 1:1, seeing as 30 mg of oral morphine is equivalent to 30 mg of oral hydrocodone. As with other opioids, use with caution in patients with renal and hepatic impairment.

#### Slide 12

Oxycodone is another oral agent and has a slightly faster onset of action of 10–15 minutes compared to the other oral opioids. The duration of action is around 3–6 hours. It is hepatically metabolized so there are similar warnings with use in patients with some liver or renal impairment.

#### Slide 13

Hydromorphone comes in both intravenous and oral formulations. It has an onset of action of 10–15 minutes and can last 3–6 hours. It is hepatically metabolized to hydromorphone-3-glucuronide (H3G), which has no analgesic or sedative effects, but could potentially have some neuroexcitatory effects. However, accumulation of this metabolite hasn’t really shown any clinical significance as of yet so it can be relatively safe to use in patients with renal impairment. Ideally, one would just start at lower and less frequent dosing. The equivalent IV dose to IV morphine is 1.5 mg.

#### Slide 14

The final opioid mentioned today is IV fentanyl. The onset of action is very rapid, usually under a minute. The duration is between 30–60 minutes. It is hepatically metabolized, but the metabolite is both inactive and non-toxic. Thus, this may be a good option if opioids are needed in a patient with liver or kidney failure. The equivalent dose to IV morphine is 0.1mg, or 100 mcg. Fentanyl is typically dosed in “mcg” so be very cognizant of the units when ordering this medication due to potential risks to patient safety.

#### Slide 15

There are some recommendations when treating patients with liver failure or insufficiency. Keep in mind, the safer opioid medication options for pain control in patients with hepatic insufficiency are IV fentanyl or hydromorphone. This is because there is a high risk of accumulation of morphine in patients with liver & renal insufficiency because the parent compound and metabolites cannot be metabolized or cleared at a normal rate, increasing risk of toxicity.

#### Slide 16

In the same way that there are preferred medications for those with hepatic insufficiency, there are also specific opioid medications that are preferred over others for patients exhibiting hemodynamic instability. Particularly, shorter-acting agents such as fentanyl are preferred. Also note that morphine actually provokes histamine release, which could exacerbate a patient’s hypotension.

#### Slide 17

When opioids are used, the lowest possible effective dosage should be prescribed to reduce risks of opioid use disorder and overdose.

However, keep in mind that when you are converting between opioid prescriptions in chronic opioid users, the dosage of the new opioid should be decreased by 25 – 50%* of the calculated morphine milligram equivalents of the current opioid regimen to avoid unintentional overdose caused by incomplete cross-tolerance and individual differences in opioid pharmacokinetics.

Also make sure to always keep in mind the big picture when treating patients with opioid medications. Take into account a patient’s age, presenting condition or chief complaint, full history, and clinical situation when prescribing opioids.

#### Slide 18

Here is an example question that shows how to use the method of morphine milligram equivalents to convert between medications, specifically oxycodone and morphine.

The case is as follows: An 80 kg cancer patient is taking 20 mg of oxycodone ER (Oxycontin) twice a day. If you were to convert this dosage to oral morphine immediate release, what is the equivalent daily dose that you would need to give to your patient to achieve the same amount of pain relief?

With the chart, you would be able to determine that 20 mg of oral oxycodone is equivalent to 30 mg of oral morphine.

In that case, next you would format your equation in the same way that is done on the screen, with the patient’s dosage of oxycodone as the numerator, over x amount of morphine and set that equal to the equivalent dosages of the medications. When you solve for X, you will see that 20x = 1,200, so X = 60 milligrams of oral morphine. Also note (as is also delineated on the infographic), when you convert between one opioid and another, remember to decrease the equivalent dosage by 25–50% to account for cross-tolerance if the patient is a chronic opioid user.

#### Slide 19

Not only can you use the morphine equivalents chart to convert between various opioids, but you can also calculate a patient’s total daily dose of opioids. This method was adapted from the CDC handout for calculating total daily dose of opioids for safer dosage.

For instance, let’s consider the cancer patient mentioned in the previous slide, but now he’s taking both Oxycontin 20 mg PO BID *and* Dilaudid 4 mg PO q4–6 hours as needed for breakthrough pain. He states to you that he usually takes 3 tablets of Dilaudid PRN a day to help control his pain.

To calculate his total daily dose of opioids, convert both total doses of oxycodone and hydromorphone to oral MMEs and add them together.

#### Slide 20

To do so, first determine the total amount of oxycodone and total amount of Dilaudid that the patient is taking. If he is taking 20 mg of oxycodone BID, that comes out to a total of 40 mg oxycodone. Additionally, because he reports taking 3 tablets of Dilaudid 4 mg as needed for breakthrough pain, he takes a total of 12 mg Dilaudid PO per day.

You can then convert both those doses to morphine milligram equivalents. You can refer to the infographic to find the conversion rates.

The conversion rate for oxycodone is 20 mg oxycodone PO to 30 mg morphine PO. By solving for x^1^, you find that 40 mg oxycodone PO equates to 60 mg of oral morphine.

The conversion rate for oral Dilaudid is 7.5 mg Dilaudid PO to 30 mg morphine PO. By solving for x^2^, you find that 12 mg Dilaudid PO equates to 48 mg of oral morphine.

You would then sum x^1^ and x^2^ and find that the patient is taking a total dose of 108 morphine milligram equivalents *daily* for pain.

#### Slide 21

Please note that the patient is *not* taking 108 morphine milligram equivalents at once for pain control. He is taking 30 equivalents each time he takes his Oxycontin, and 16 equivalents each time he takes his Dilaudid as needed.

In conclusion, the patient is taking 30 morphine milligram equivalents twice a day through his Oxycontin regimen, plus an additional 16 morphine milligram equivalents, three times a day, through his Dilaudid prescription to address his breakthrough pain.

#### Slide 22

Now, we can apply the infographic information and conversion techniques that we’ve learned to a real-world clinical scenario in the emergency department.

Keep in mind, the patient was on a chronic opioid regimen that amounted to 108 morphine milligram equivalents per day.

If that same patient came to see you in the emergency room, complaining of new right arm pain after falling and breaking his humerus (a non-operative fracture), how would you go about treating his pain?

Consider, what initial dose do you give him for pain control?

#### Slide 23

There are three different routes that you can choose to take when addressing acute pain in a patient who is opioid-tolerant.

The first option is to convert the patient’s home oral regimen to an IV dose, then give a one-time IV dose as needed and monitor the patient’s response.

The second option would be to continue the patient on his/her home oral regimen and add an IV option as necessary.

The last option is to increase the patient’s home oral dosage. For example, you could consider increasing to 15 mg of oxycodone if a patient was taking 10 mg at home.

#### Slide 24

So, for this patient with the humerus fracture, we previously calculated that he is on a total of 108 morphine milligram equivalents daily at home.

In the emergency department, we could consider transitioning him to an IV medication. In this slide, we show two examples of IV medications that you could consider and show how to convert the patient’s dosage to equivalent doses of IV morphine or IV Dilaudid.

As you can see on the slide, 30 mg of oral morphine is equal to 10 mg of IV morphine. So solving for that equation, we see that 108 mg of morphine PO is equal to 36 mg of morphine IV.

You can also consider using Dilaudid to treat the patient’s pain. 30 mg of oral morphine is equal to 1.5 mg of IV dilaudid, so 108 mg of morphine PO is equal to 5.4 mg of IV Dilaudid.

#### Slide 25

Note, for an opioid-naive patient in the ED, the recommended one-time dose of IV morphine used to address pain is 0.05–0.1 mg/kg.

Using that guideline, an 80 kg patient would be given between 4 mg and 8 mg of IV morphine q4–6 hours as needed for pain relief.

#### Slide 26

However, this patient has a past medical history of cancer and therefore is a chronic opioid user, so you must consider tolerance. These patients will require more pain control.

As you can see on this slide, to determine a one time IV pain medication dose, you can take the dose of the new IV medication that is equivalent to the amount that the patient is taking at home and divide by 4. In this case, we are choosing to use either morphine IV or Dilaudid IV. Doing so results in a single dose of 9 mg morphine IV or 1.35 mg Dilaudid IV. Therefore, you can consider a one-time dose of 8 mg morphine IV or 1 mg Dilaudid IV to treat the patient’s pain. The doses have been decreased slightly in order to adjust for cross-tolerance in this chronic opioid user. After giving that single dose, you can monitor the patient’s pain and continue to titrate the medication accordingly. If your ED prefers to schedule doses, you can schedule as q4–6 instead of the one-time dose.

#### Slide 27

In general, if a patient is experiencing pain, providers will often give a single dose of opioids in the ED, reassess the patient for pain relief, and titrate subsequent doses as necessary. The first dose is dependent on the institution and whether the patient is opioid-naive or opioid-tolerant.

The morphine equianalgesic chart can be used to convert to an appropriate dose of oral pain medication when a patient is being discharged. In chronic opioid users, total MME can be calculated and the home regimen may be adjusted.

#### Slide 28

This table shows some initial opioid doses for acute pain treatment and administration frequencies for a standard adult patient. It is split into two columns: opioid naive versus opioid tolerant. Please note that these doses merely act as suggestions and do not preclude clinical judgment nor institution practice. To define opioid tolerant, we used the FDA definition of a patient who has been taking at least 60 mg oral morphine, 30 mg of oral oxycodone, 8 mg of oral hydromorphone or an equianalgesic dose of another oral opioid on a daily basis for more than 1 week.

#### Slide 29

Let’s go over a few more clinical scenarios. In this first scenario, a 54-year-old man is brought in by EMS as a trauma following a motor vehicle accident. Past medical history is significant for a gunshot wound to his right leg thirty years ago, for which he takes oxycodone daily. After administration of 100 mcg of fentanyl, the patient states that he has had no pain relief. What medication should be used to help control his pain, and at what dosage?

#### Slide 30

Consider that the patient’s pain was not relieved at all by the 100 mcg of fentanyl. In this case, you could use the morphine milligram equivalents chart and choose a different opioid medication to test out whether a switch in medication could help address his pain. According to the infographic, 0.1 mg of fentanyl IV is equivalent to 1.5 mg of Dilaudid IV.

When you convert 100 mcg of fentanyl IV to Dilaudid IV, you find that the equivalent dose is 1.5 mg of Dilaudid IV.

In that case, you could try giving the patient the equivalent dosage of 1.5 mg of Dilaudid IV and monitor his response. Clinically, you could also consider repeating the same dose of 100 mcg of IV fentanyl and monitor his response. Throughout the patient’s ED stay, you will continue to judge his clinical response, and titrate dosage based on patient relief.

Upon discharge, you can then sum up the total morphine milligram equivalents needed to control his pain and create a chronic pain regimen for him.

#### Slide 31

The second clinical scenario is as follows: A 47-year-old woman comes to the ED for abdominal pain. You give her one dose of 4 mg IV morphine for pain. When you reassess her, she continues to complain of 10/10 pain and is asking for stronger medication. You decide to give her a dose of Dilaudid. What dose of Dilaudid must you exceed in order to achieve better pain relief, if 4 mg IV morphine did not alleviate her pain?

#### Slide 32

The first step would be to calculate the equivalent dosage of 4 mg of morphine IV to Dilaudid IV. According to the infographic, 10 mg of morphine IV is equivalent to 1.5 mg of Dilaudid IV. Therefore, x equals 0.6 mg of Dilaudid IV. So, you must exceed 0.6 mg of Dilaudid IV when providing the patient with a stronger medication in order to achieve better pain relief.

#### Slide 33

Thank you for your time and attention!

### Appendix 3 Opioid Equianalgesic Chart

Please see associated PDF file

### Appendix 4 CDC Calculating Total Daily Dose of Opioids for Safer Dosage

Please see associated PDF file or visit https://www.cdc.gov/opioids/providers/prescribing/guideline.html

### Appendix 5 Morphine Equivalents Survey Questions

1. What is the equianalgesic conversion of morphine 10 mg IV to Hydromorphone IV?
  - 1.5 mg
  - 2.5 mg
  - 3.5 mg
  - 4.5 mg
2. A patient is receiving a dose of fentanyl 100 mcg IV. What is the equivalent dose of morphine?
  - 1 mg IV
  - 2 mg IV
  - 10 mg IV
  - 20 mg IV
3. Which of the following drugs has the longest onset of action?
  - Hydromorphone PO
  - Oxycodone PO
  - Morphine IV
  - Hydrocodone PO
4. Which of the following drugs has the shortest onset of action?
  - Morphine PO
  - Morphine IV
  - Hydromorphone PO
  - Oxycodone PO
5. Which conversion is equivalent to hydromorphone 0.5 mg IV?
  - Fentanyl 1 mg IV
  - Hydrocodone 10 mg PO
  - Morphine 5 mg IV
  - Oxycodone 2 mg PO
6. What is the onset of action of fentanyl when given IV?
  - Less than 60 seconds
  - Greater than 60 seconds but less than 1 hour
  - 1–3 hours
  - 3–4 hours
7. How does the onset of action of morphine PO compare to oxycodone PO?
  - Morphine PO has a faster onset than oxycodone PO
  - Morphine PO has the same onset of action as oxycodone PO
  - Morphine PO has a slower onset than oxycodone PO
8. Choose the correct equianalgesic conversion for 100 mg Oxycodone PO.
  - Hydromorphone 35 mg PO
  - Hydromorphone 40 mg PO
  - Morphine 50 mg IV
  - Morphine 160 mg PO
9. Which of the following statements is false?
  - Morphine IV is more potent than oral hydromorphone
  - Hydromorphone IV is more potent than oral morphine
  - Oral oxycodone is less potent than oral hydromorphone
  - Morphine IV is more potent than oral oxycodone
10. A patient is receiving hydromorphone 30 mg PO but you want to switch medications to morphine PO. Taking into account cross-tolerance, what is an accepted dosage of morphine PO to prescribe?
  - Morphine 150 mg PO
  - Morphine 120 mg PO
  - Morphine 80 mg PO
  - Morphine 50 mg PO

### Appendix 6 Morphine Equivalents Survey Answers

1. What is the equianalgesic conversion of morphine 10 mg IV to Hydromorphone IV?
  - **1.5 mg**
  - 2.5 mg
  - 3.5 mg
  - 4.5 mg
2. A patient is receiving a dose of fentanyl 100 mcg IV. What is the equivalent dose of morphine?
  - 1 mg IV
  - 2 mg IV
  - **10 mg IV**
  - 20 mg IV
3. Which of the following drugs has the longest onset of action?
  - Hydromorphone PO
  - Oxycodone PO
  - Morphine IV
  - **Hydrocodone PO**
4. Which of the following drugs has the shortest onset of action?
  - Morphine PO
  - **Morphine IV**
  - Hydromorphone PO
  - Oxycodone PO
5. Which conversion is equivalent to hydromorphone 0.5 mg IV?
  - Fentanyl 1 mg IV
  - **Hydrocodone 10 mg PO**
  - Morphine 5 mg IV
  - Oxycodone 2 mg PO
6. What is the onset of action of fentanyl when given IV?
  - **Less than 60 seconds**
  - Greater than 60 seconds but less than 1 hour
  - 1–3 hours
  - 3–4 hours
7. How does the onset of action of morphine PO compare to oxycodone PO?
  - Morphine PO has a faster onset than oxycodone PO
  - Morphine PO has the same onset of action as oxycodone PO
  - **Morphine PO has a slower onset than oxycodone PO**
8. Choose the correct equianalgesic conversion for 100 mg Oxycodone PO.
  - Hydromorphone 35 mg PO
  - Hydromorphone 40 mg PO
  - **Morphine 50 mg IV**
  - Morphine 160 mg PO
9. Which of the following statements is false?
  - **Morphine IV is more potent than oral hydromorphone**
  - Hydromorphone IV is more potent than oral morphine
  - Oral oxycodone is less potent than oral hydromorphone
  - Morphine IV is more potent than oral oxycodone
10. A patient is receiving hydromorphone 30 mg PO but you want to switch medications to morphine PO. Taking into account cross-tolerance, what is an accepted dosage of morphine PO to prescribe?
  - Morphine 150 mg PO
  - Morphine 120 mg PO
  - **Morphine 80 mg PO**
  - Morphine 50 mg PO
