# Supplementary material for: Morphine Equianalgesic Dose Chart in the Emergency Department
Source: J Educ Teach Emerg Med. 2022 Jul 15;7(3):L1–L20. doi: 10.21980/J8RD29 (PMC10332699; doi:10.21980/J8RD29)
Supplement: Supplementary file 1 — Please see associated PowerPoint file [file jetem-7-3-l1-appendix1.pptx]

## Slide 1
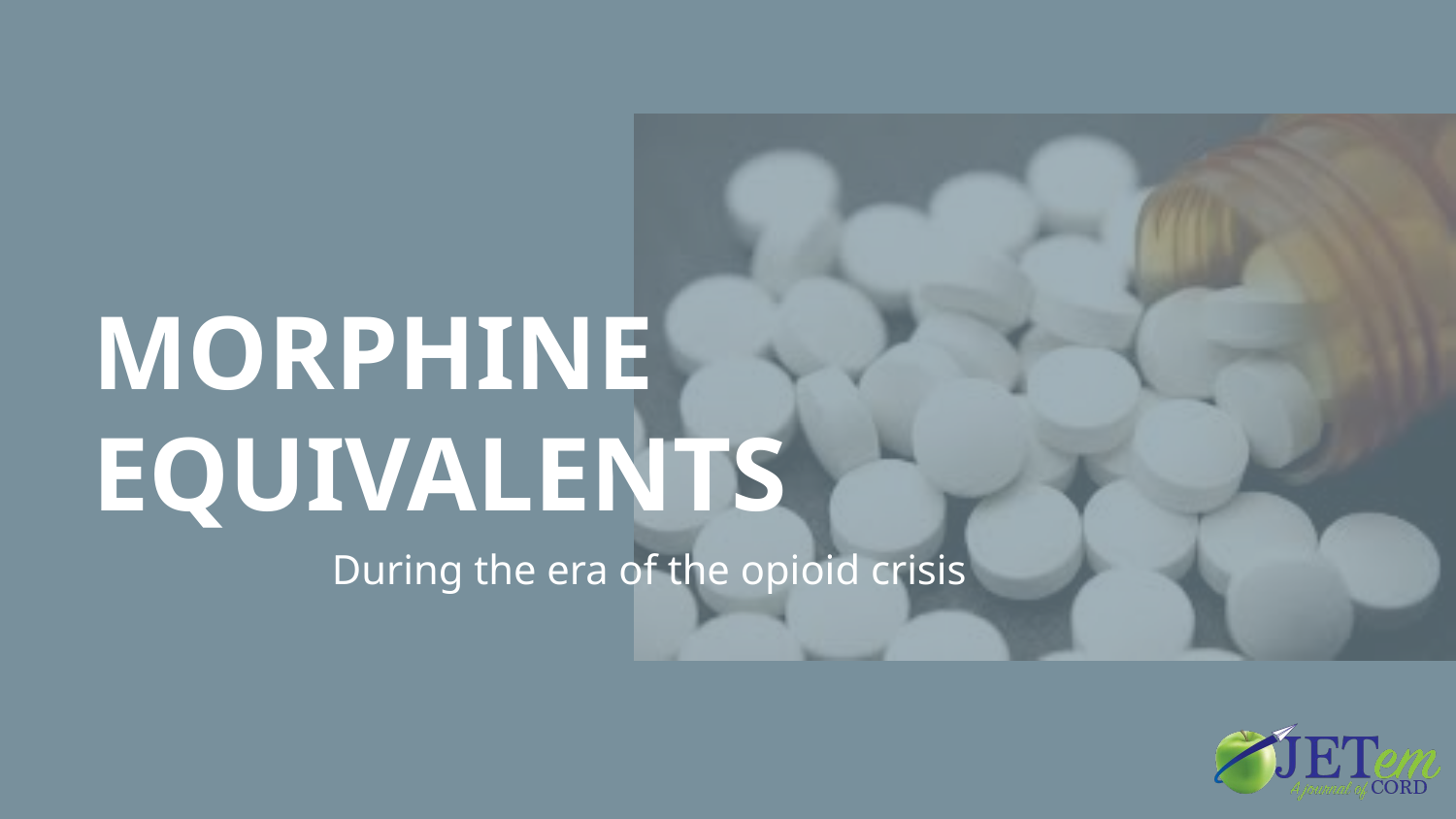

# MORPHINE
EQUIVALENTS
During the era of the opioid crisis

## Slide 2
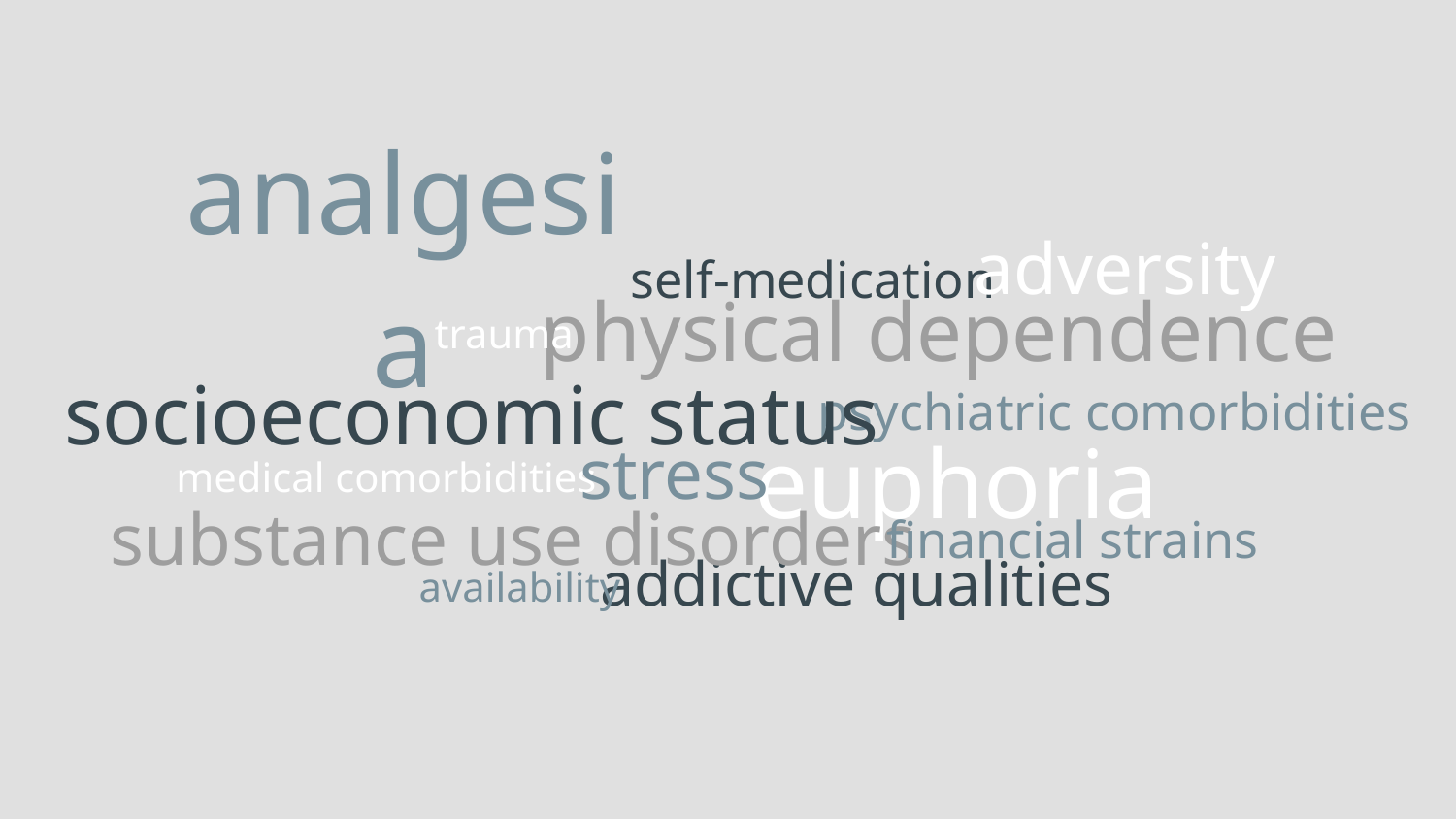

analgesia
self-medication
adversity
# physical dependence
trauma
socioeconomic status
psychiatric comorbidities
euphoria
stress
medical comorbidities
substance use disorders
addictive qualities
financial strains
availability

## Slide 3
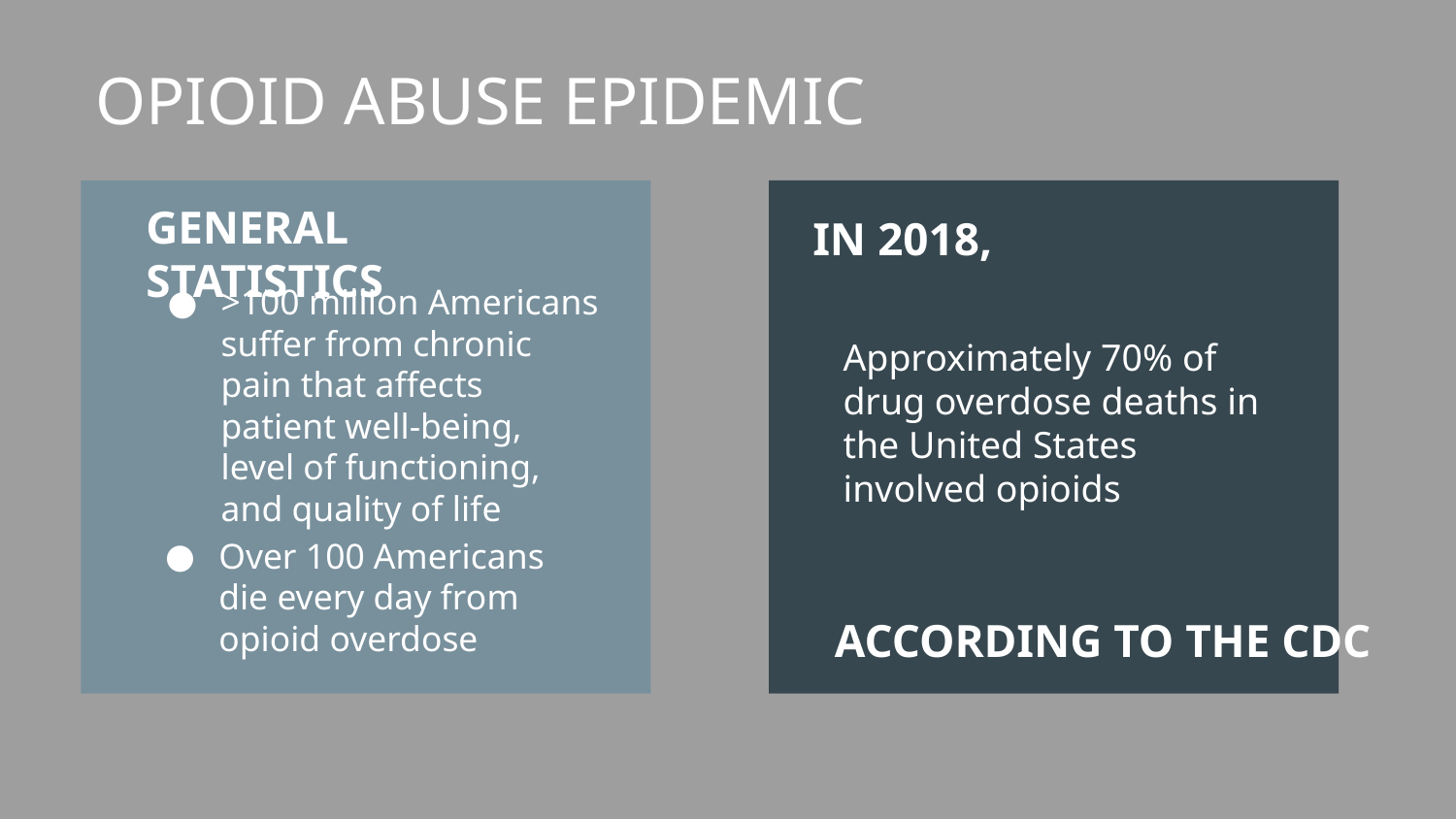

# OPIOID ABUSE EPIDEMIC
GENERAL STATISTICS
IN 2018,
>100 million Americans suffer from chronic pain that affects patient well-being, level of functioning, and quality of life
Approximately 70% of drug overdose deaths in the United States involved opioids
Over 100 Americans die every day from opioid overdose
ACCORDING TO THE CDC

## Slide 4
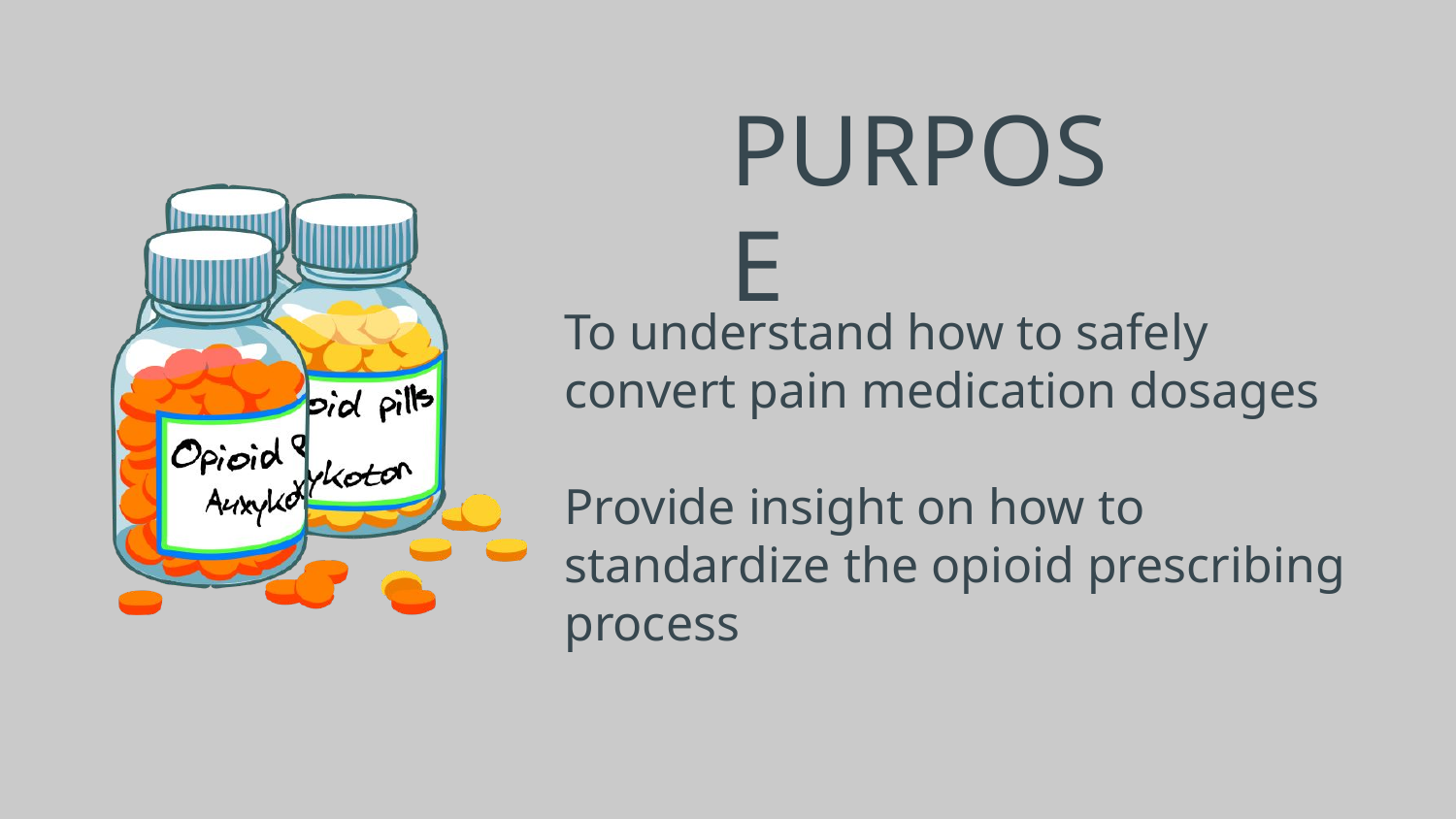

# PURPOSE
To understand how to safely convert pain medication dosages
Provide insight on how to standardize the opioid prescribing process

## Slide 5
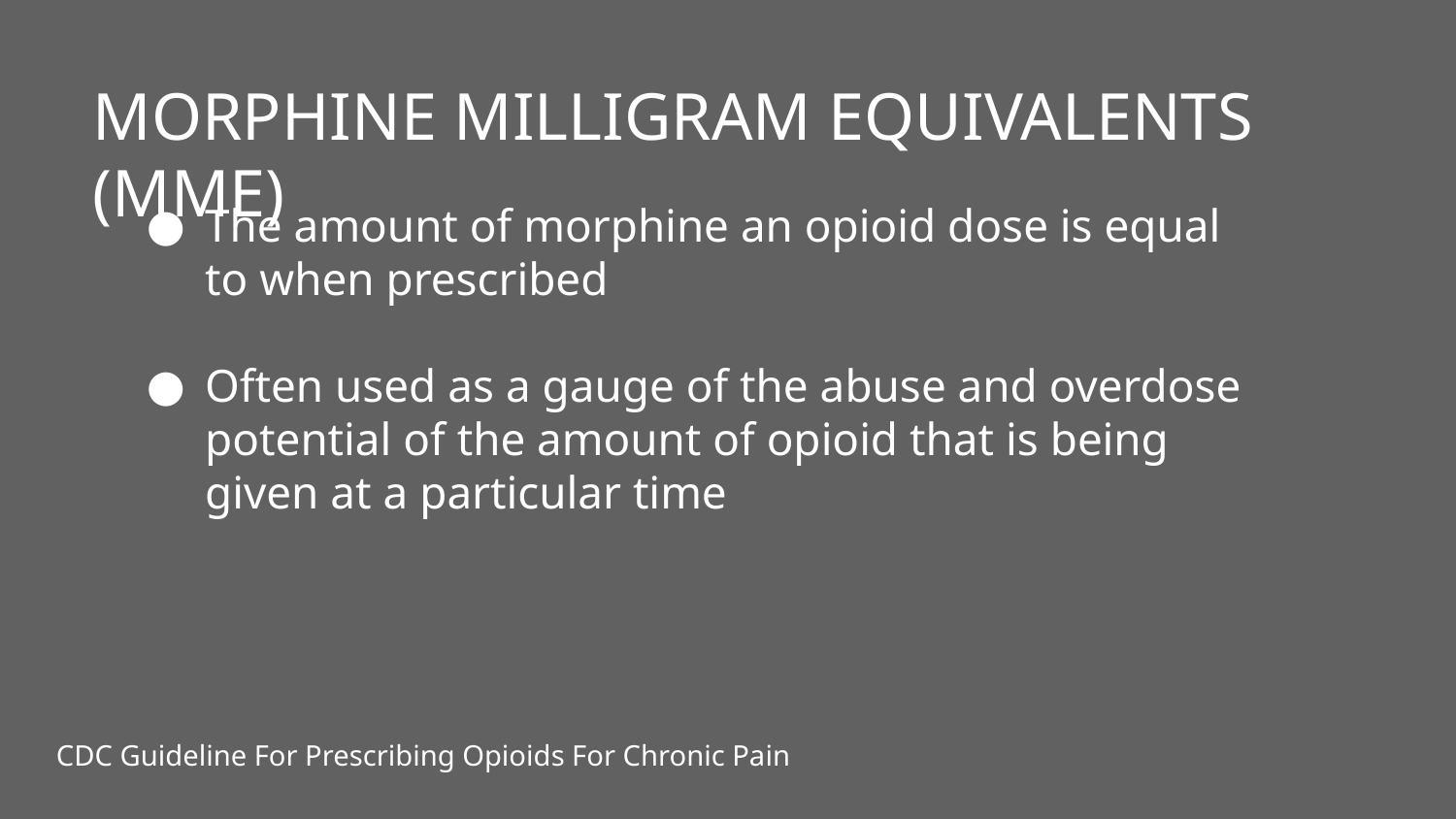

MORPHINE MILLIGRAM EQUIVALENTS (MME)
# The amount of morphine an opioid dose is equal to when prescribed
Often used as a gauge of the abuse and overdose potential of the amount of opioid that is being given at a particular time
CDC Guideline For Prescribing Opioids For Chronic Pain

## Slide 6
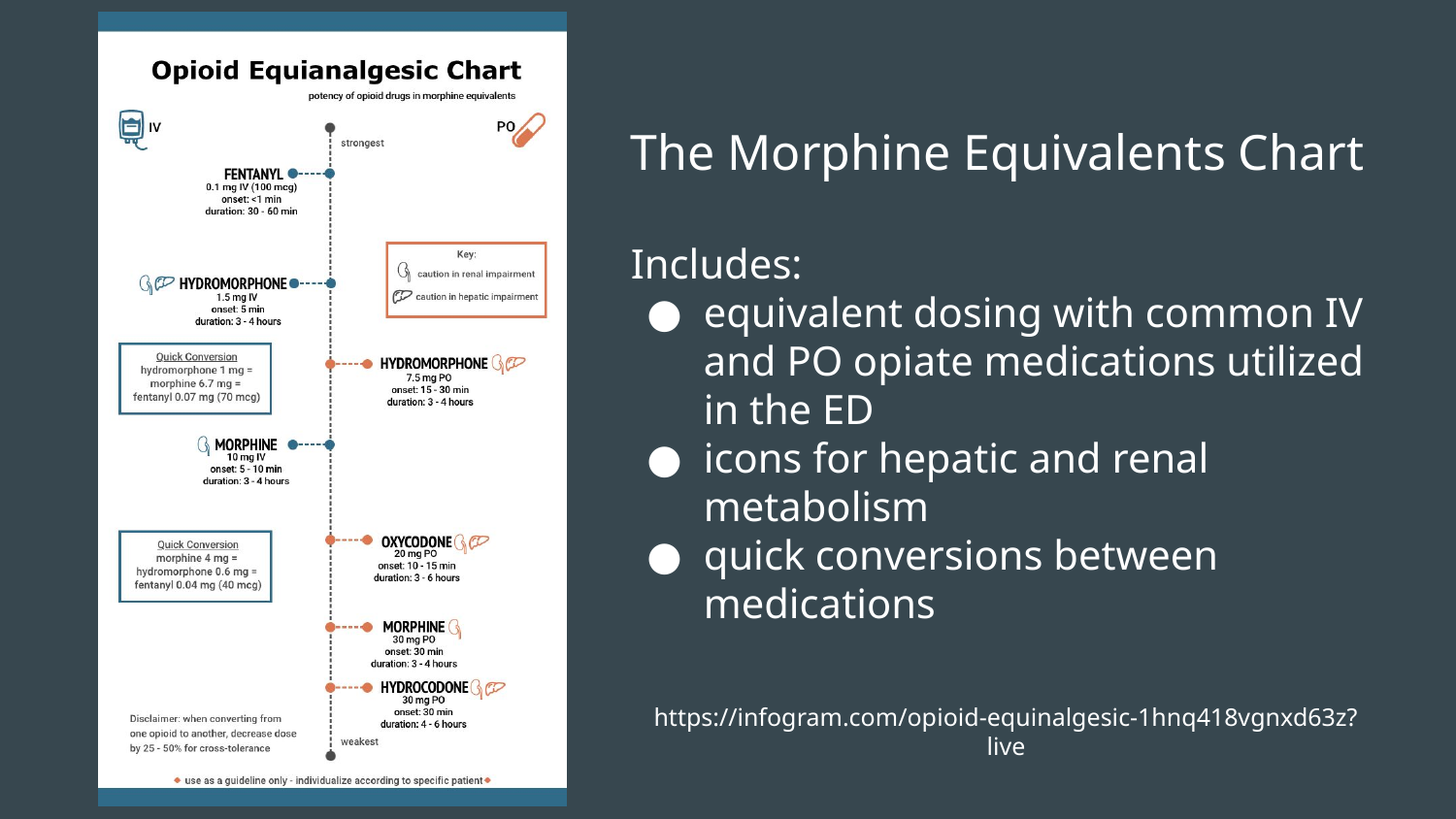

The Morphine Equivalents Chart
Includes:
equivalent dosing with common IV and PO opiate medications utilized in the ED
icons for hepatic and renal metabolism
quick conversions between medications
https://infogram.com/opioid-equinalgesic-1hnq418vgnxd63z?live

## Slide 7
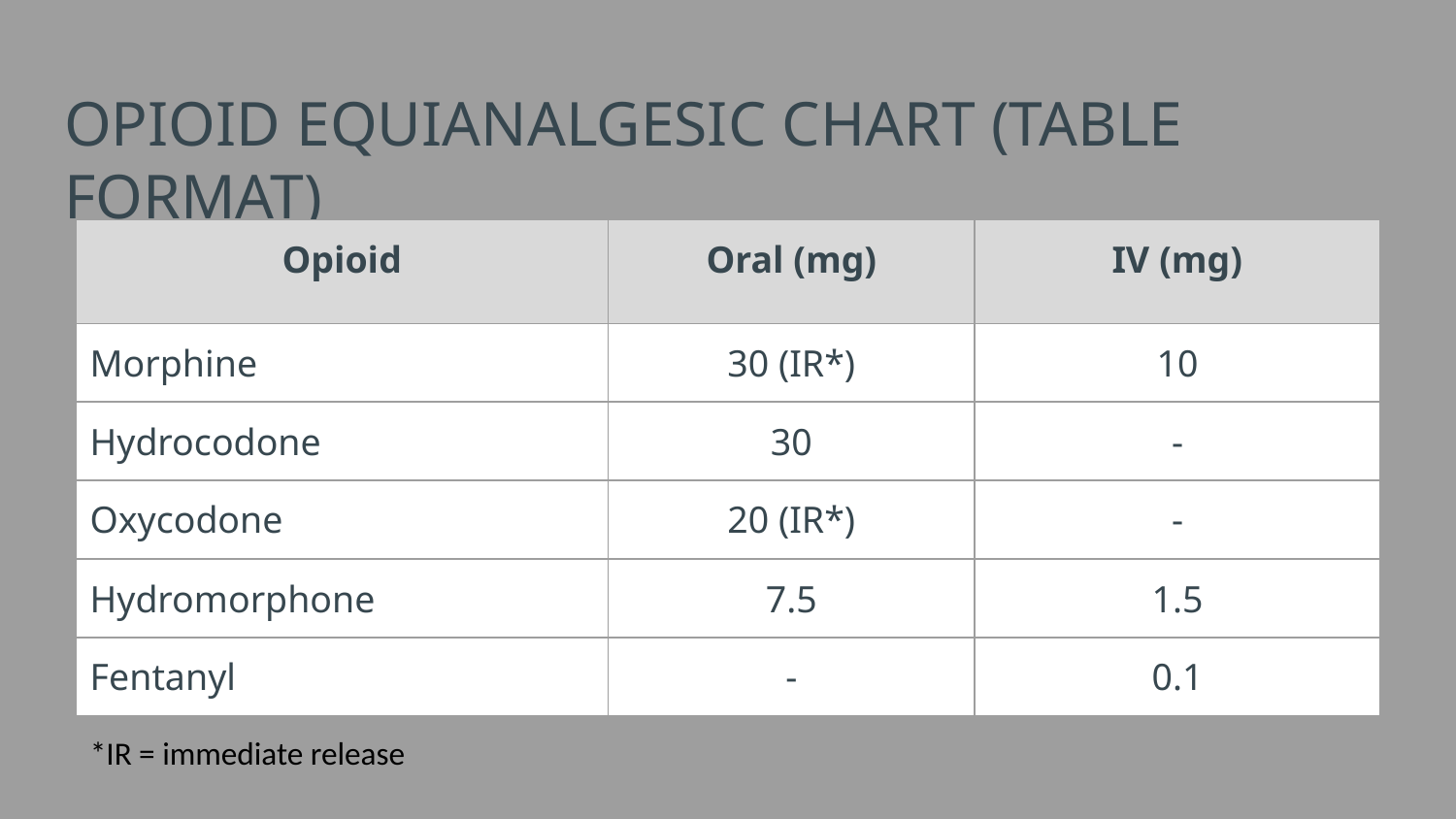

# OPIOID EQUIANALGESIC CHART (TABLE FORMAT)
| Opioid | Oral (mg) | IV (mg) |
| --- | --- | --- |
| Morphine | 30 (IR\*) | 10 |
| Hydrocodone | 30 | - |
| Oxycodone | 20 (IR\*) | - |
| Hydromorphone | 7.5 | 1.5 |
| Fentanyl | - | 0.1 |
*IR = immediate release

## Slide 8
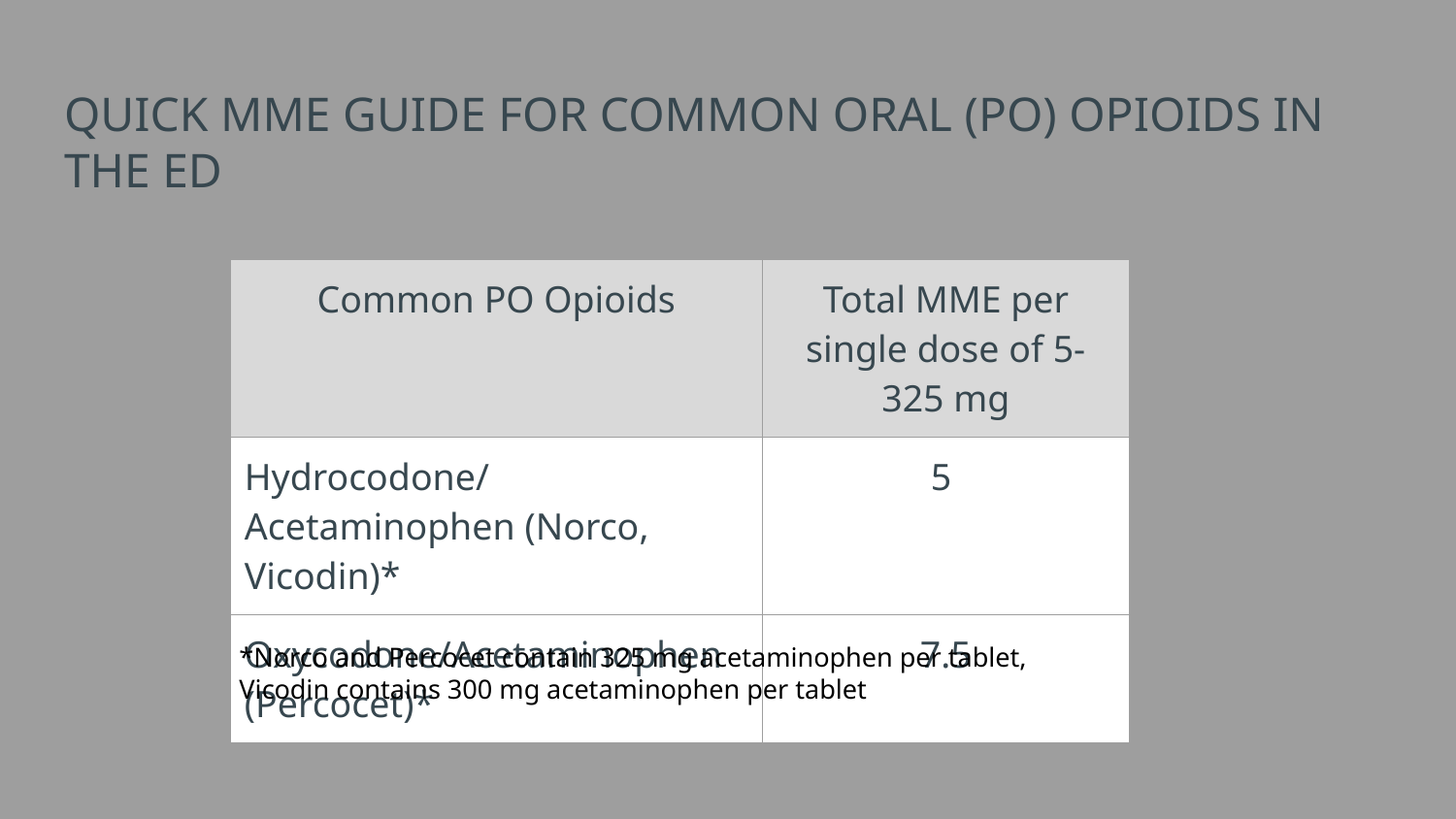

# QUICK MME GUIDE FOR COMMON ORAL (PO) OPIOIDS IN THE ED
| Common PO Opioids | Total MME per single dose of 5-325 mg |
| --- | --- |
| Hydrocodone/Acetaminophen (Norco, Vicodin)\* | 5 |
| Oxycodone/Acetaminophen (Percocet)\* | 7.5 |
*Norco and Percocet contain 325 mg acetaminophen per tablet, Vicodin contains 300 mg acetaminophen per tablet

## Slide 9
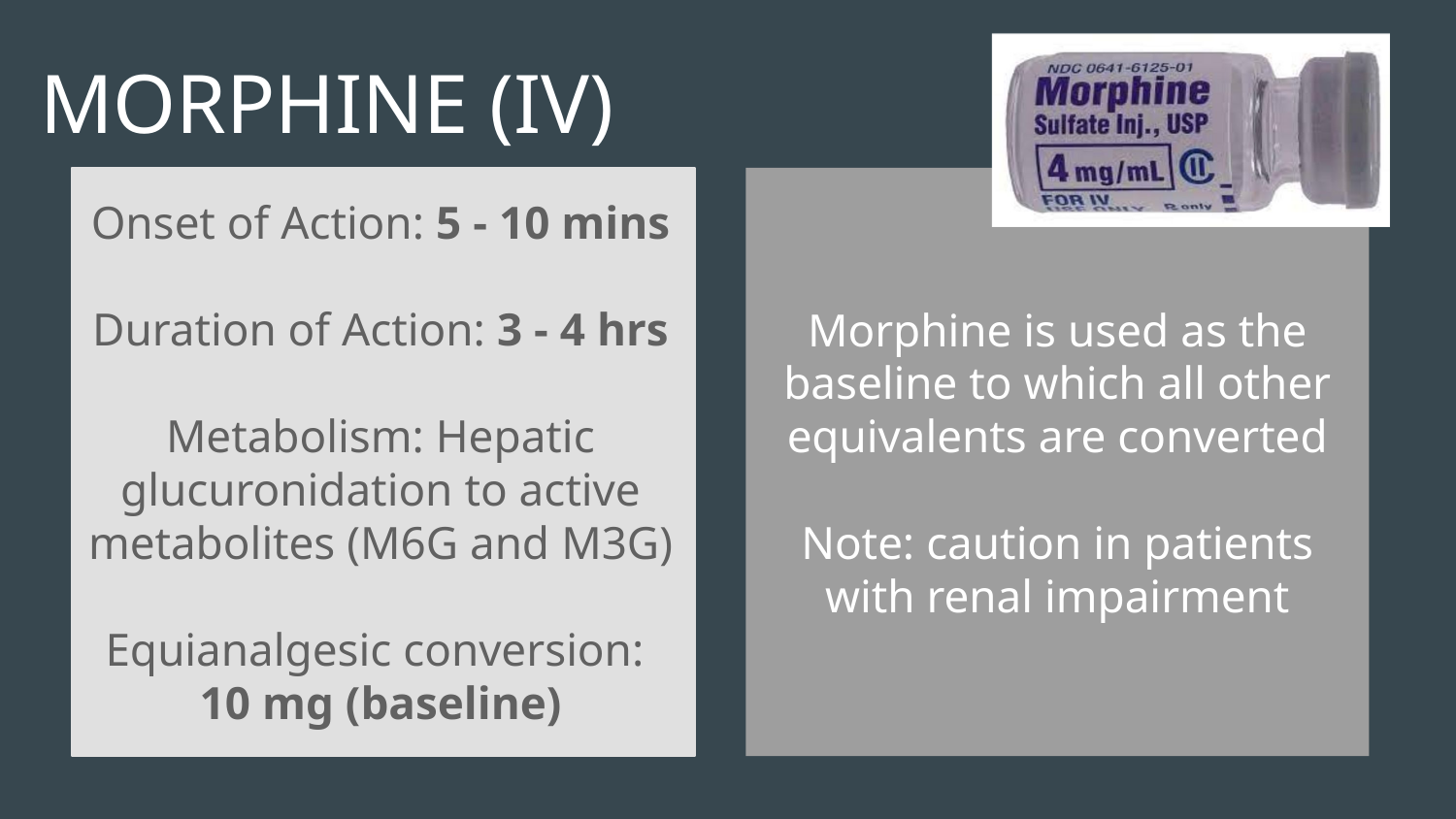

MORPHINE (IV)
Onset of Action: 5 - 10 mins
Duration of Action: 3 - 4 hrs
Metabolism: Hepatic glucuronidation to active metabolites (M6G and M3G)
Equianalgesic conversion:
10 mg (baseline)
Morphine is used as the baseline to which all other equivalents are converted
Note: caution in patients with renal impairment

## Slide 10
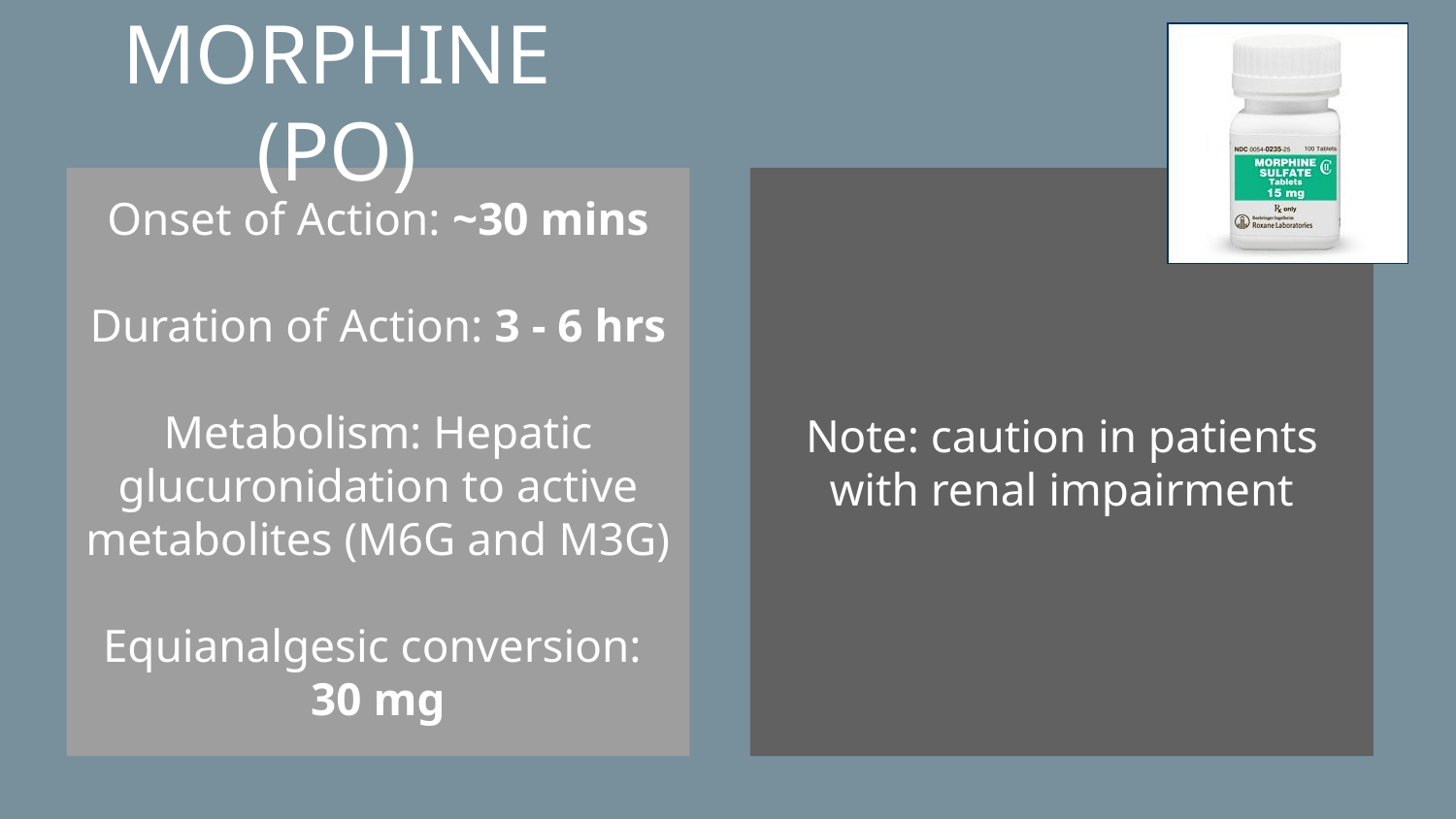

# MORPHINE (PO)
Onset of Action: ~30 mins
Duration of Action: 3 - 6 hrs
Metabolism: Hepatic glucuronidation to active metabolites (M6G and M3G)
Equianalgesic conversion:
30 mg
Note: caution in patients with renal impairment

## Slide 11
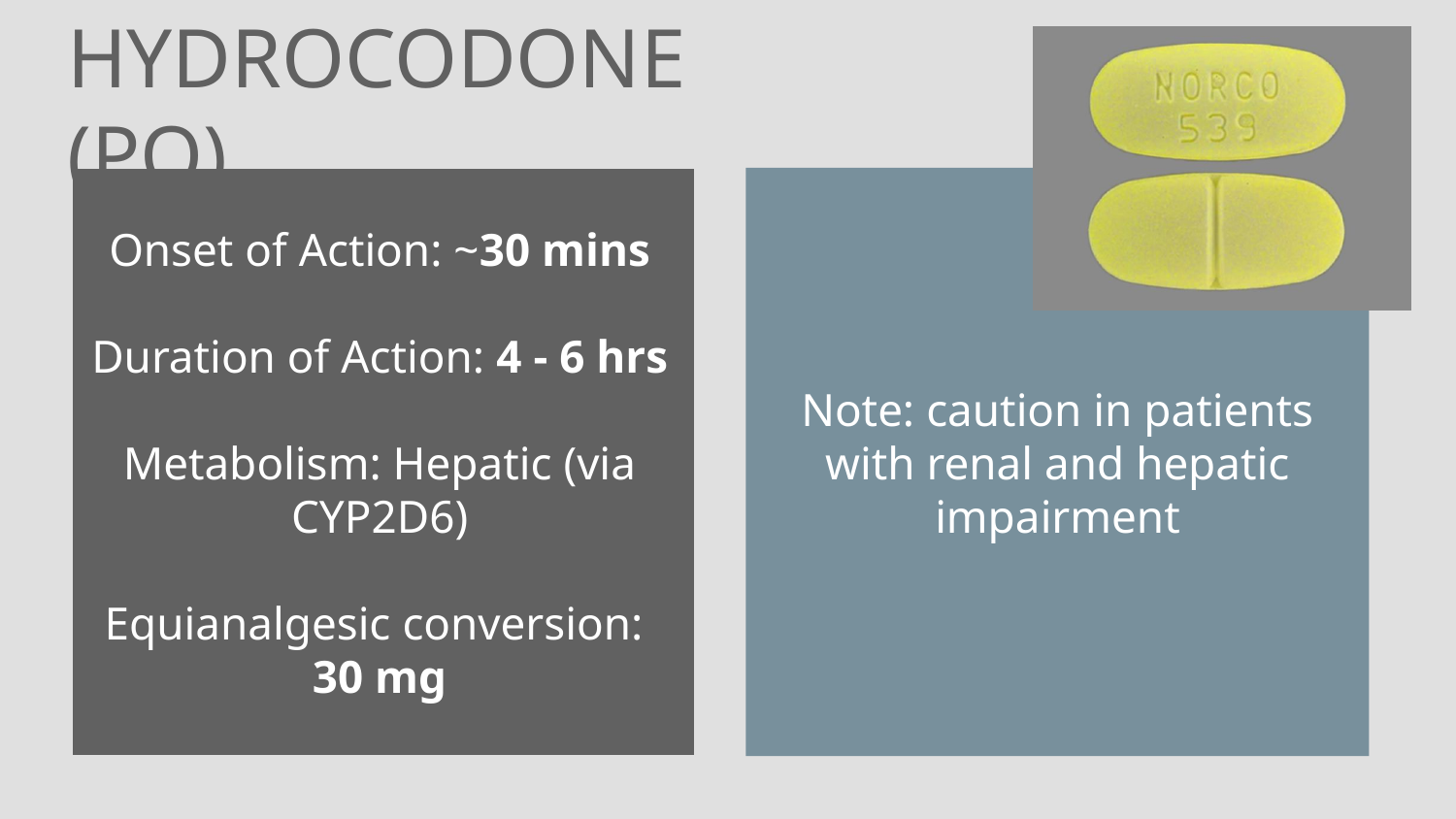

HYDROCODONE (PO)
Onset of Action: ~30 mins
Duration of Action: 4 - 6 hrs
Metabolism: Hepatic (via CYP2D6)
Equianalgesic conversion:
30 mg
# Note: caution in patients with renal and hepatic impairment

## Slide 12
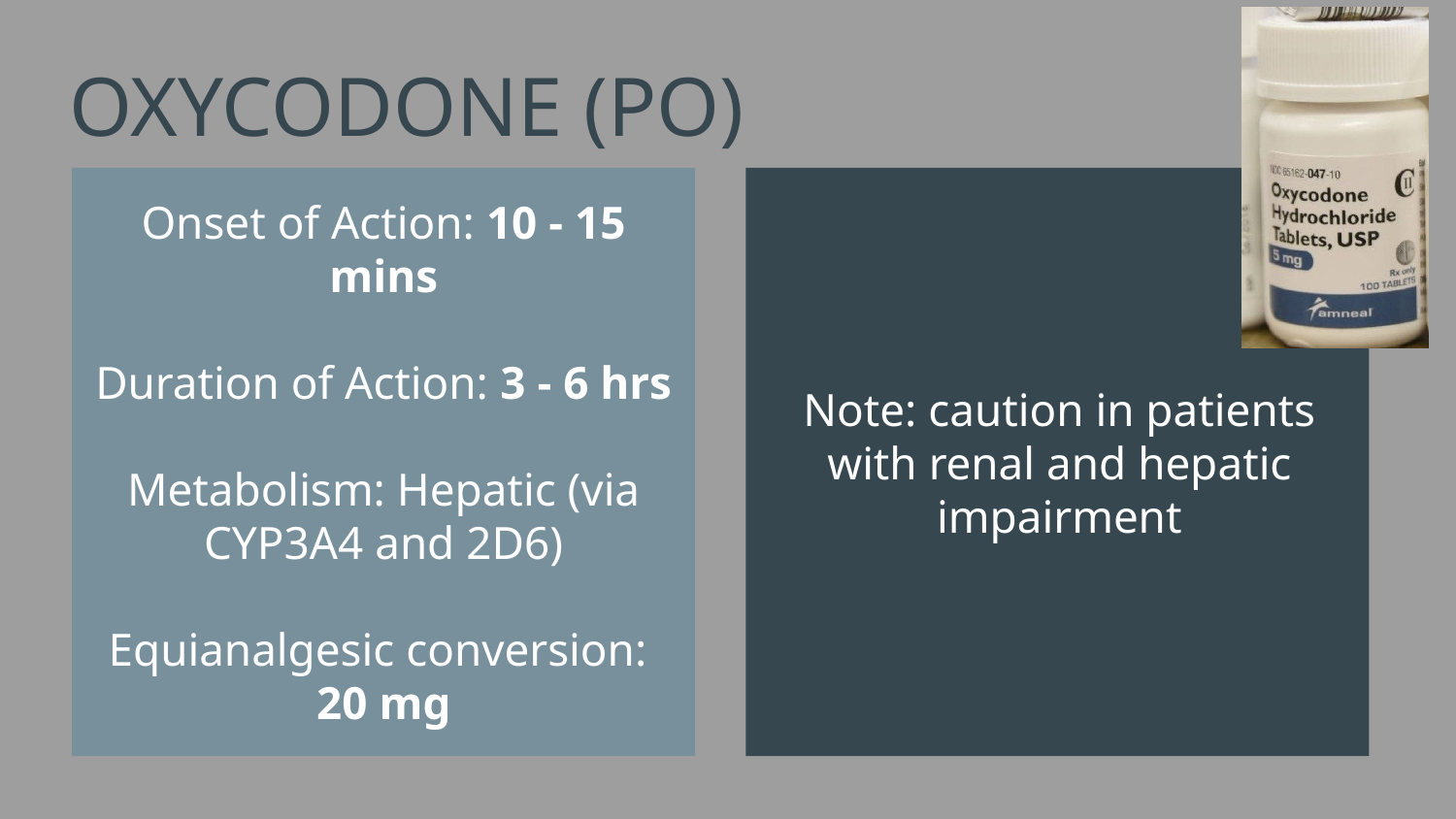

OXYCODONE (PO)
Onset of Action: 10 - 15 mins
Duration of Action: 3 - 6 hrs
Metabolism: Hepatic (via CYP3A4 and 2D6)
Equianalgesic conversion:
20 mg
# Note: caution in patients with renal and hepatic impairment

## Slide 13
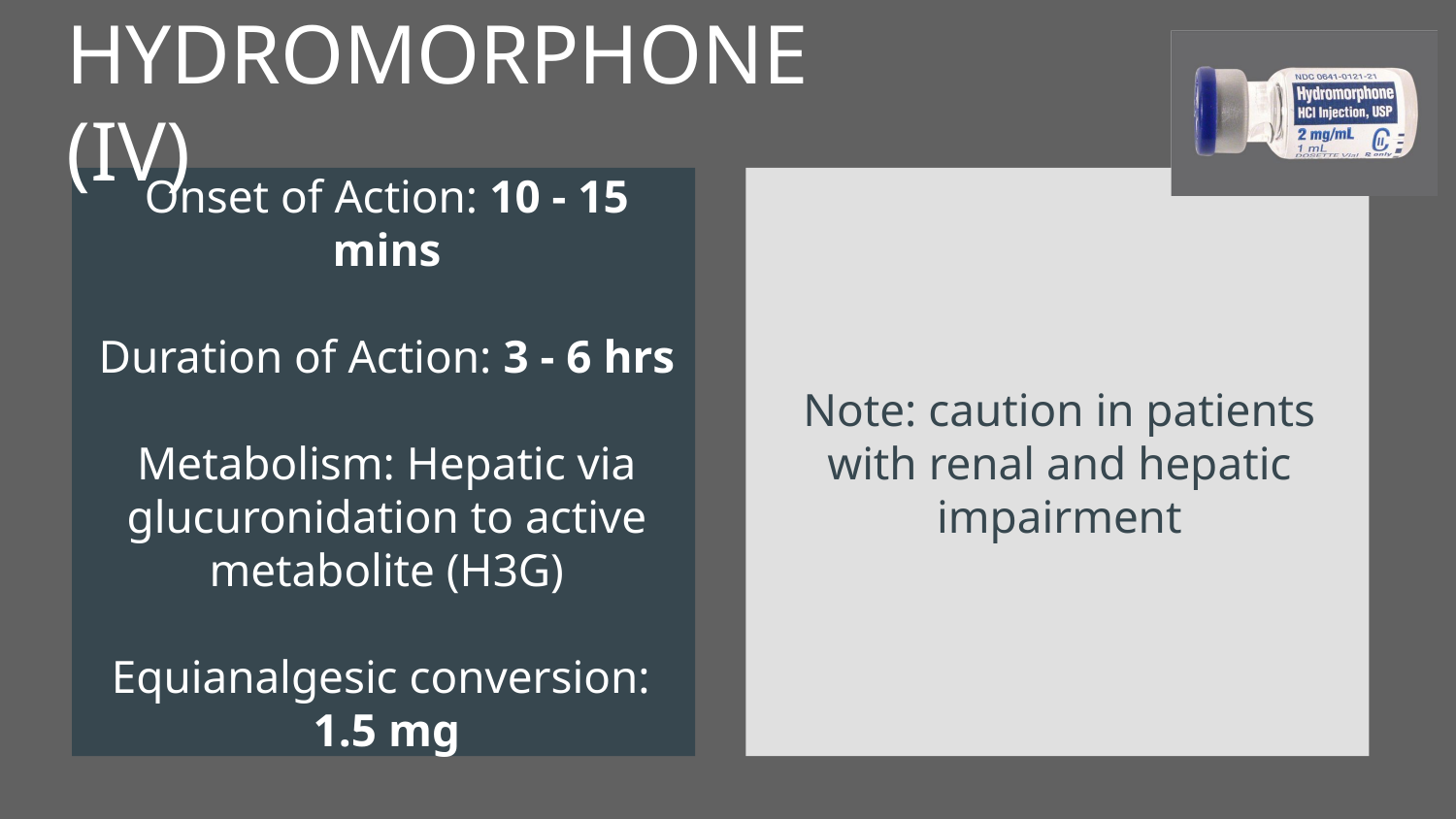

HYDROMORPHONE (IV)
Onset of Action: 10 - 15 mins
Duration of Action: 3 - 6 hrs
Metabolism: Hepatic via glucuronidation to active metabolite (H3G)
Equianalgesic conversion:
1.5 mg
# Note: caution in patients with renal and hepatic impairment

## Slide 14
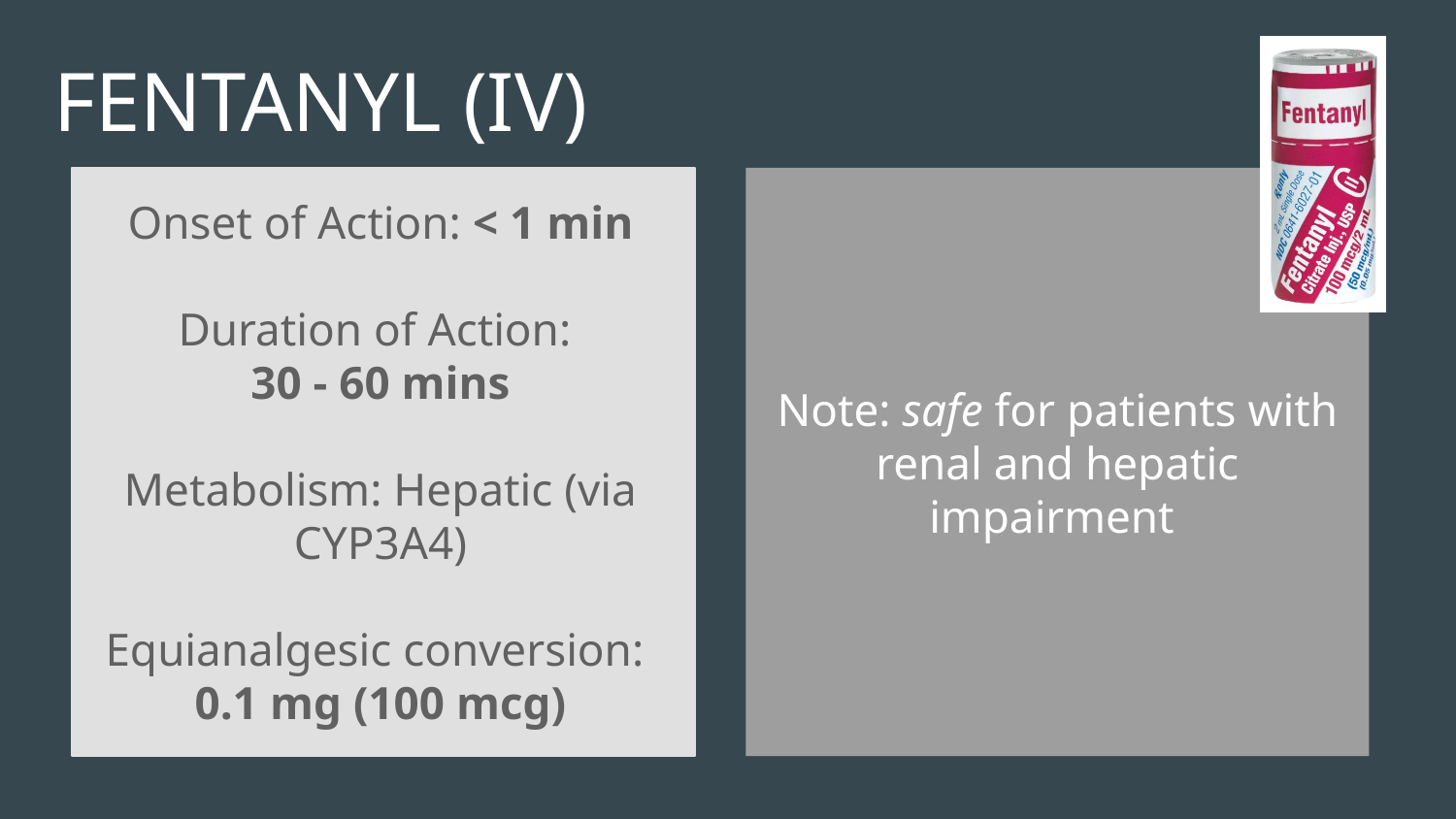

FENTANYL (IV)
Onset of Action: < 1 min
Duration of Action:
30 - 60 mins
Metabolism: Hepatic (via CYP3A4)
Equianalgesic conversion:
0.1 mg (100 mcg)
Note: safe for patients with renal and hepatic impairment

## Slide 15
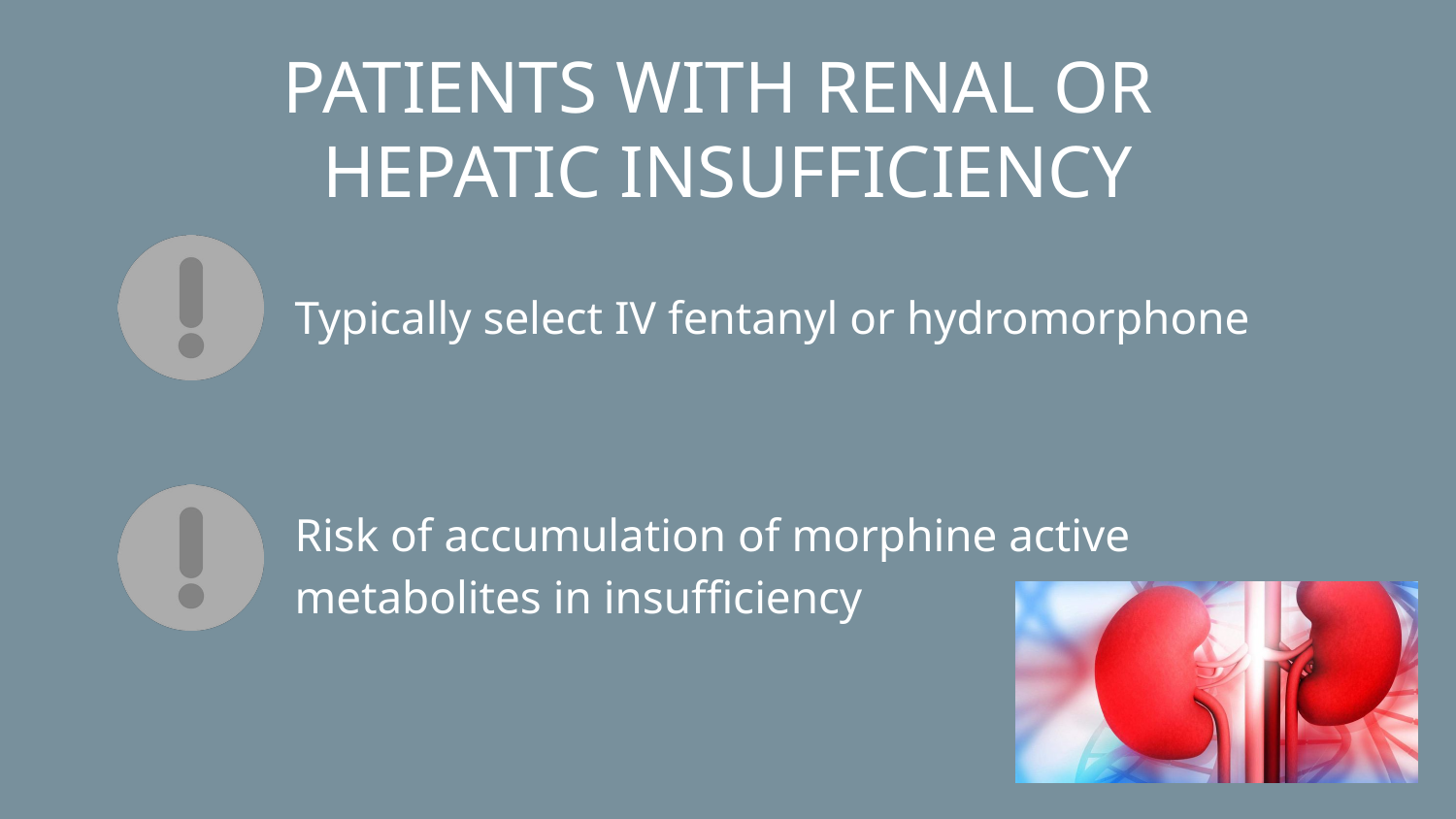

# PATIENTS WITH RENAL OR
HEPATIC INSUFFICIENCY
Typically select IV fentanyl or hydromorphone
Risk of accumulation of morphine active metabolites in insufficiency

## Slide 16
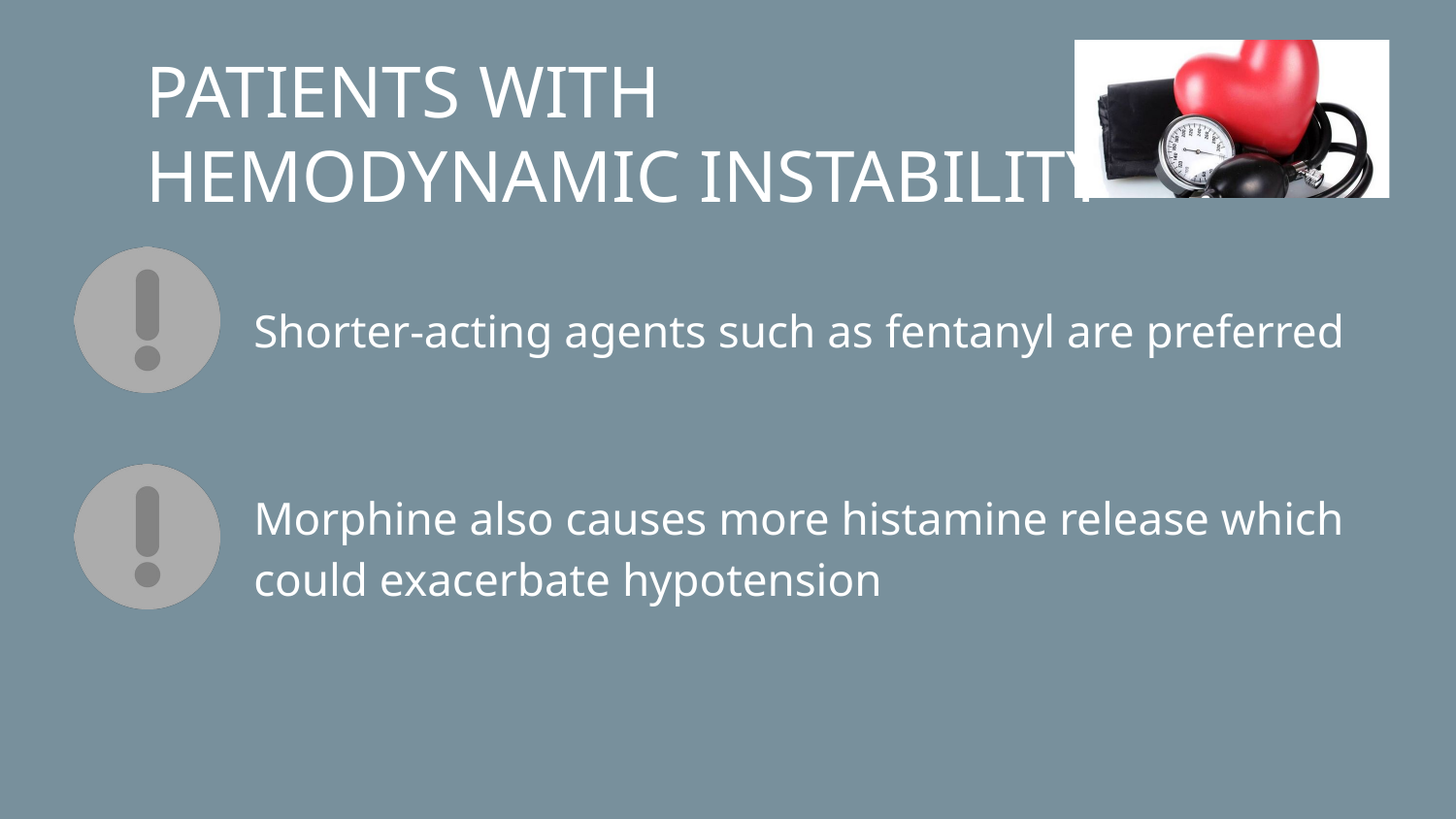

# PATIENTS WITH
HEMODYNAMIC INSTABILITY
Shorter-acting agents such as fentanyl are preferred
Morphine also causes more histamine release which could exacerbate hypotension

## Slide 17
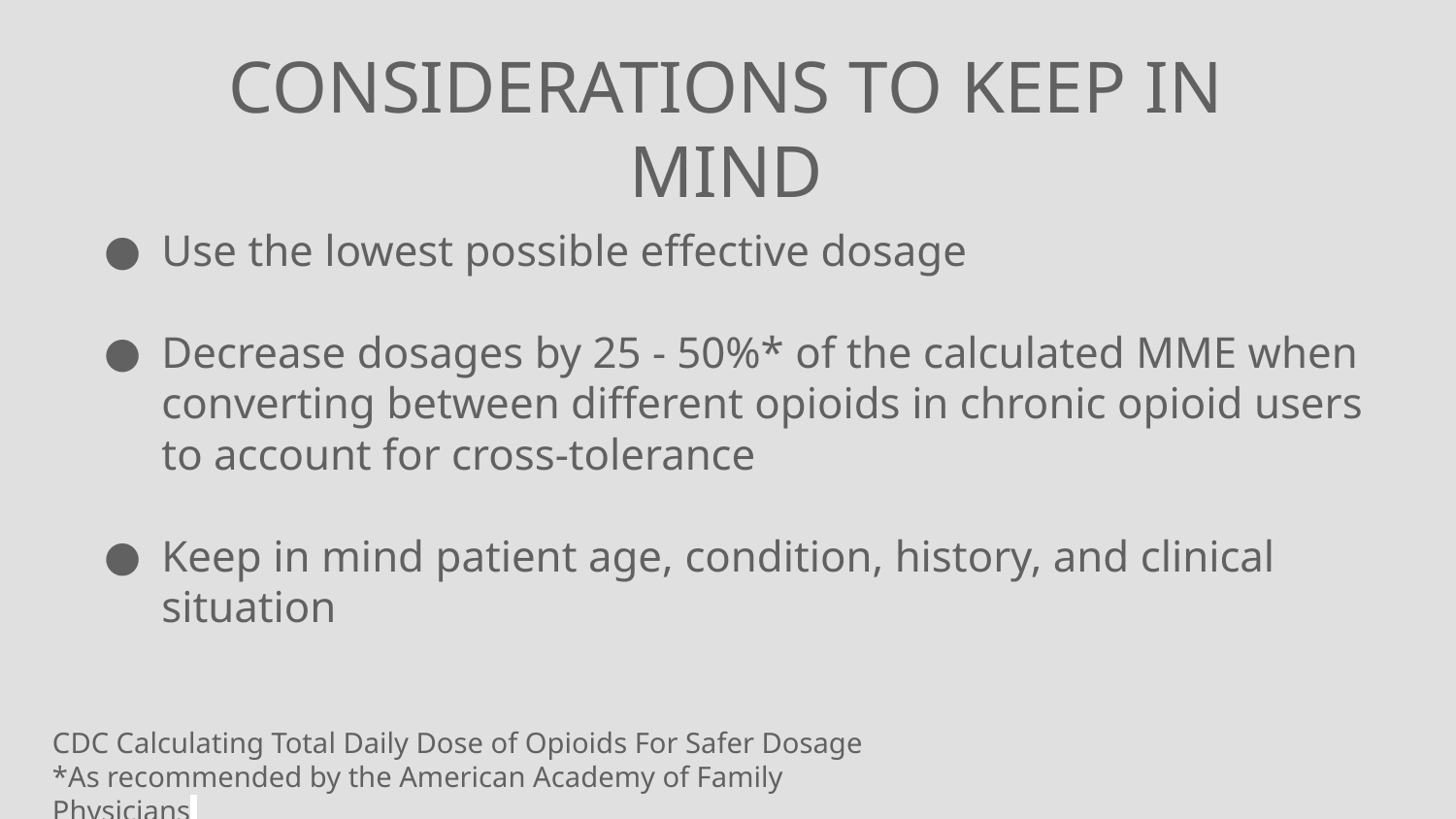

# CONSIDERATIONS TO KEEP IN MIND
Use the lowest possible effective dosage
Decrease dosages by 25 - 50%* of the calculated MME when converting between different opioids in chronic opioid users to account for cross-tolerance
Keep in mind patient age, condition, history, and clinical situation
CDC Calculating Total Daily Dose of Opioids For Safer Dosage
*As recommended by the American Academy of Family Physicians

## Slide 18
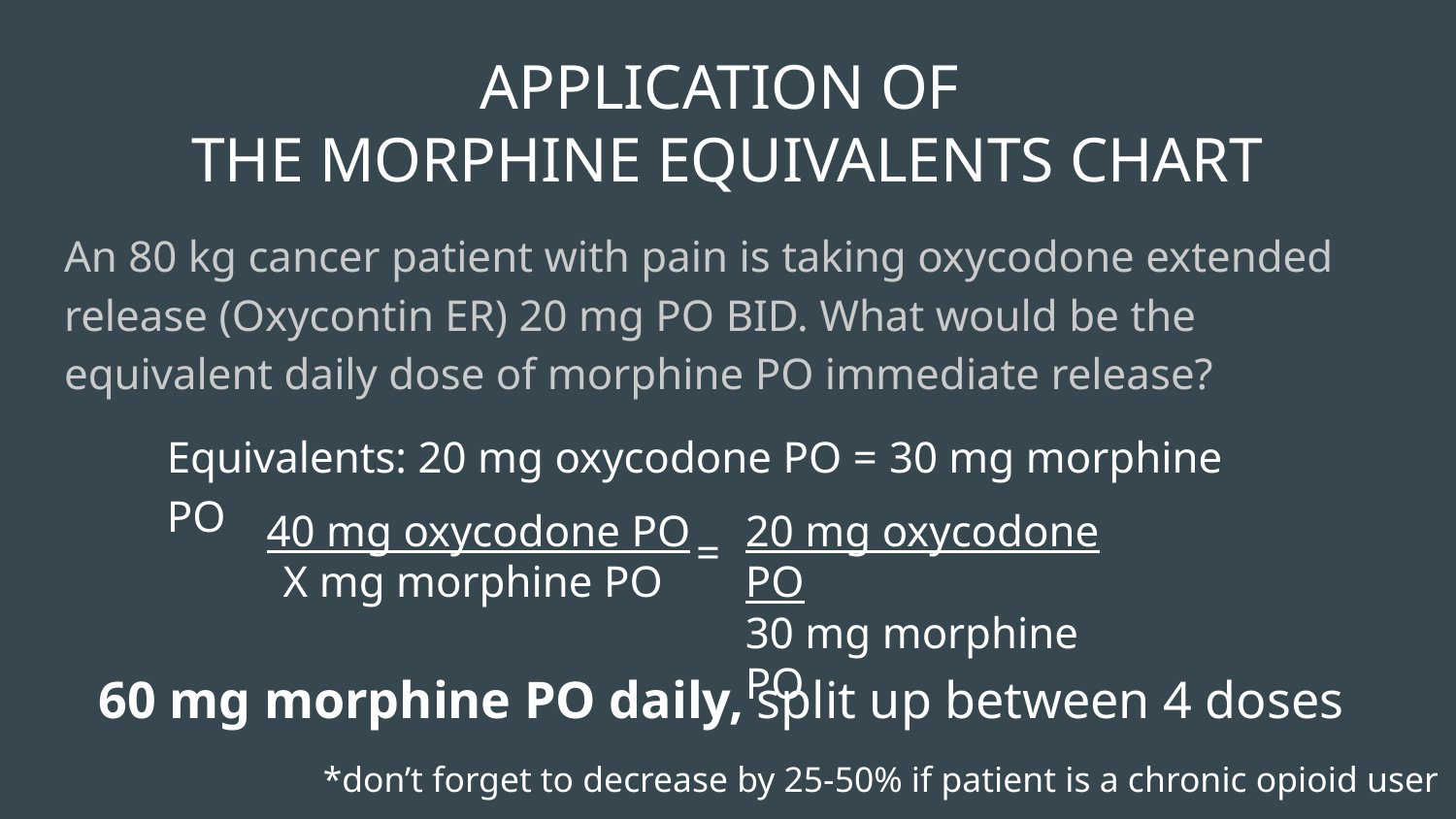

# APPLICATION OF
THE MORPHINE EQUIVALENTS CHART
An 80 kg cancer patient with pain is taking oxycodone extended release (Oxycontin ER) 20 mg PO BID. What would be the equivalent daily dose of morphine PO immediate release?
Equivalents: 20 mg oxycodone PO = 30 mg morphine PO
40 mg oxycodone PO
X mg morphine PO
20 mg oxycodone PO
30 mg morphine PO
=
60 mg morphine PO daily, split up between 4 doses
*don’t forget to decrease by 25-50% if patient is a chronic opioid user

## Slide 19
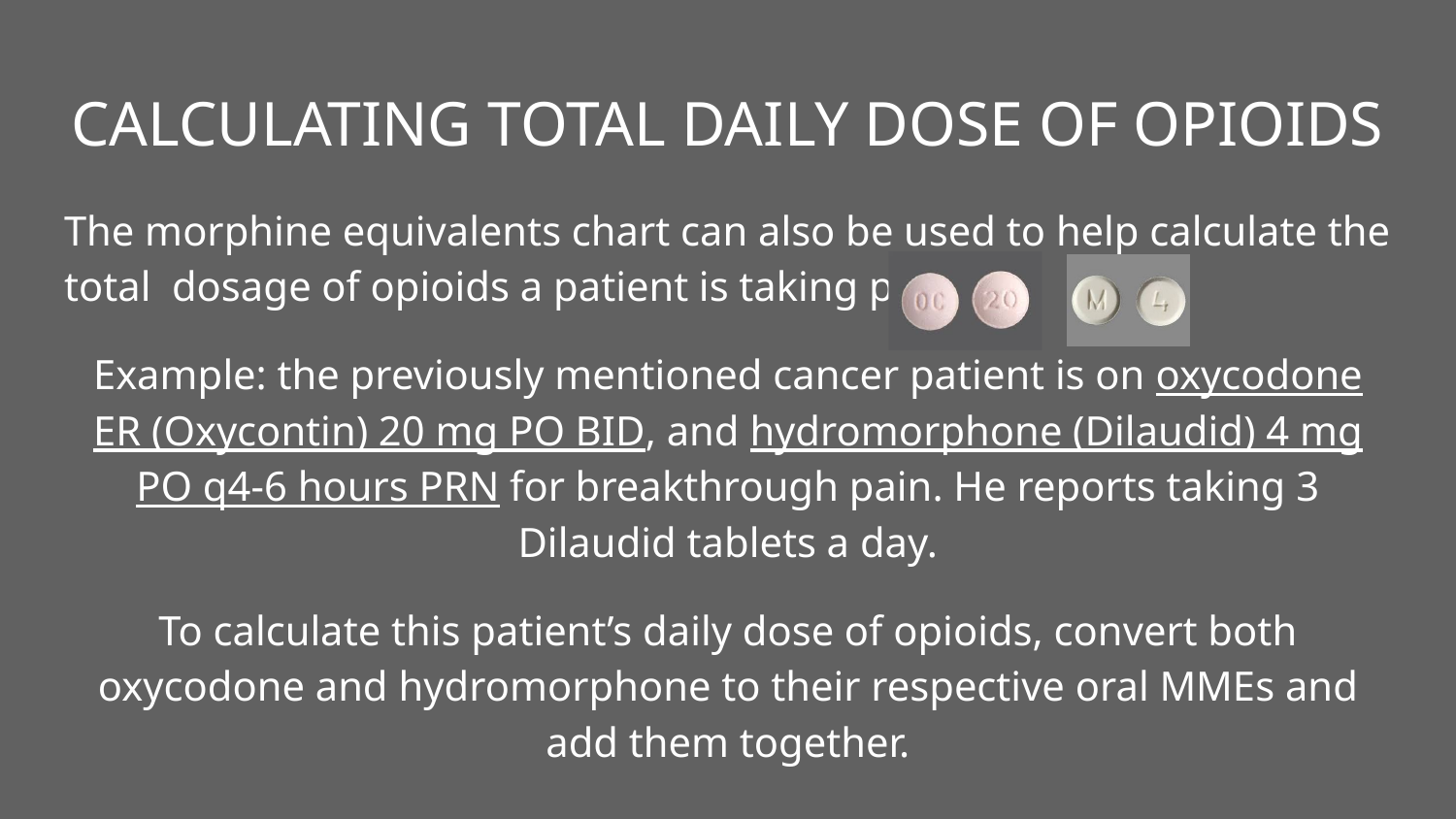

# CALCULATING TOTAL DAILY DOSE OF OPIOIDS
The morphine equivalents chart can also be used to help calculate the total dosage of opioids a patient is taking per day.
Example: the previously mentioned cancer patient is on oxycodone ER (Oxycontin) 20 mg PO BID, and hydromorphone (Dilaudid) 4 mg PO q4-6 hours PRN for breakthrough pain. He reports taking 3 Dilaudid tablets a day.
To calculate this patient’s daily dose of opioids, convert both oxycodone and hydromorphone to their respective oral MMEs and add them together.

## Slide 20
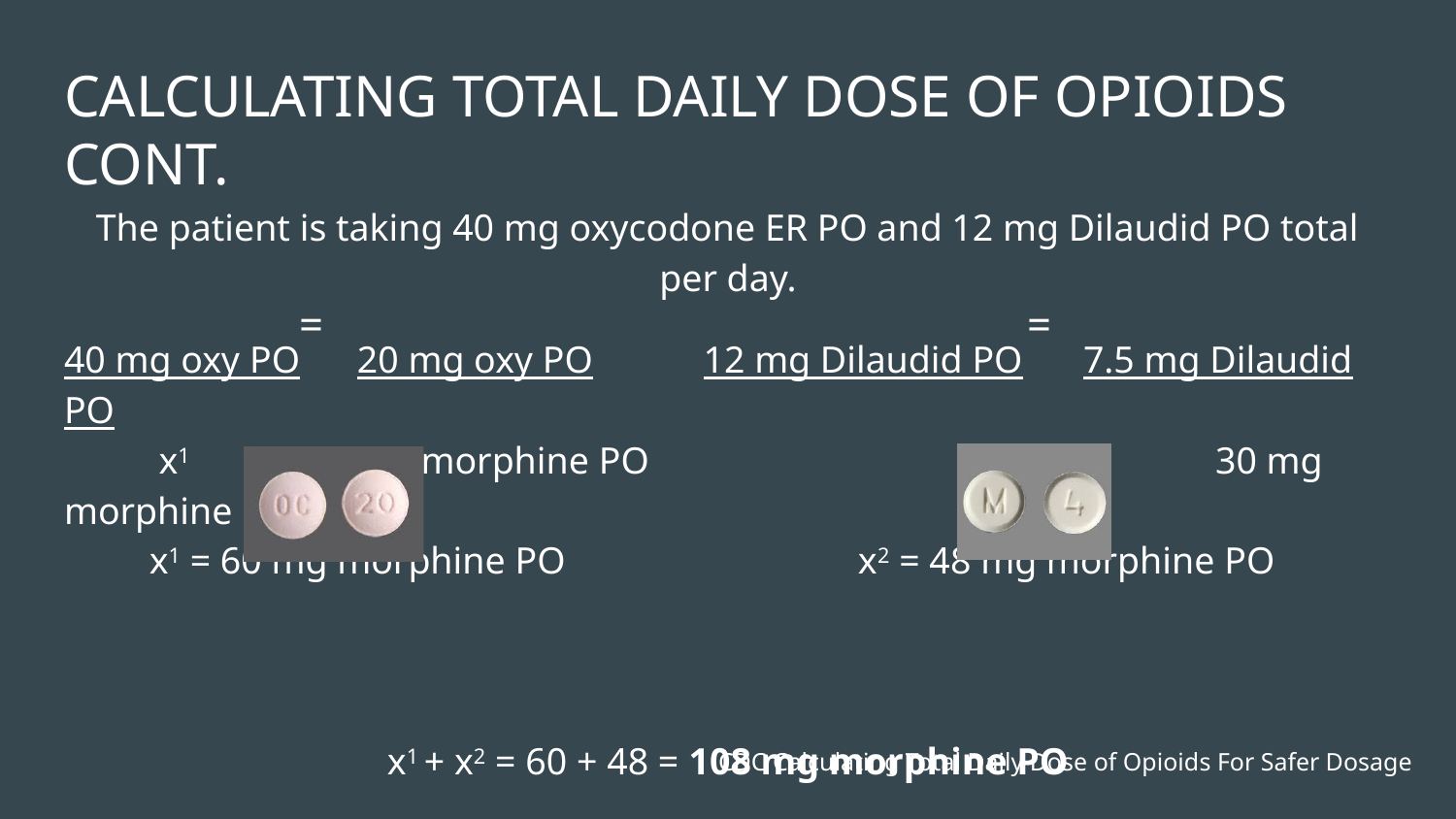

# CALCULATING TOTAL DAILY DOSE OF OPIOIDS CONT.
The patient is taking 40 mg oxycodone ER PO and 12 mg Dilaudid PO total per day.
40 mg oxy PO 20 mg oxy PO 	 12 mg Dilaudid PO	7.5 mg Dilaudid PO
 x1 	 30 mg morphine PO		 x2	 30 mg morphine PO
 x1 = 60 mg morphine PO		 x2 = 48 mg morphine PO
x1 + x2 = 60 + 48 = 108 mg morphine PO
The patient is taking a total of 108 MMEs daily for pain.
=
=
CDC Calculating Total Daily Dose of Opioids For Safer Dosage

## Slide 21
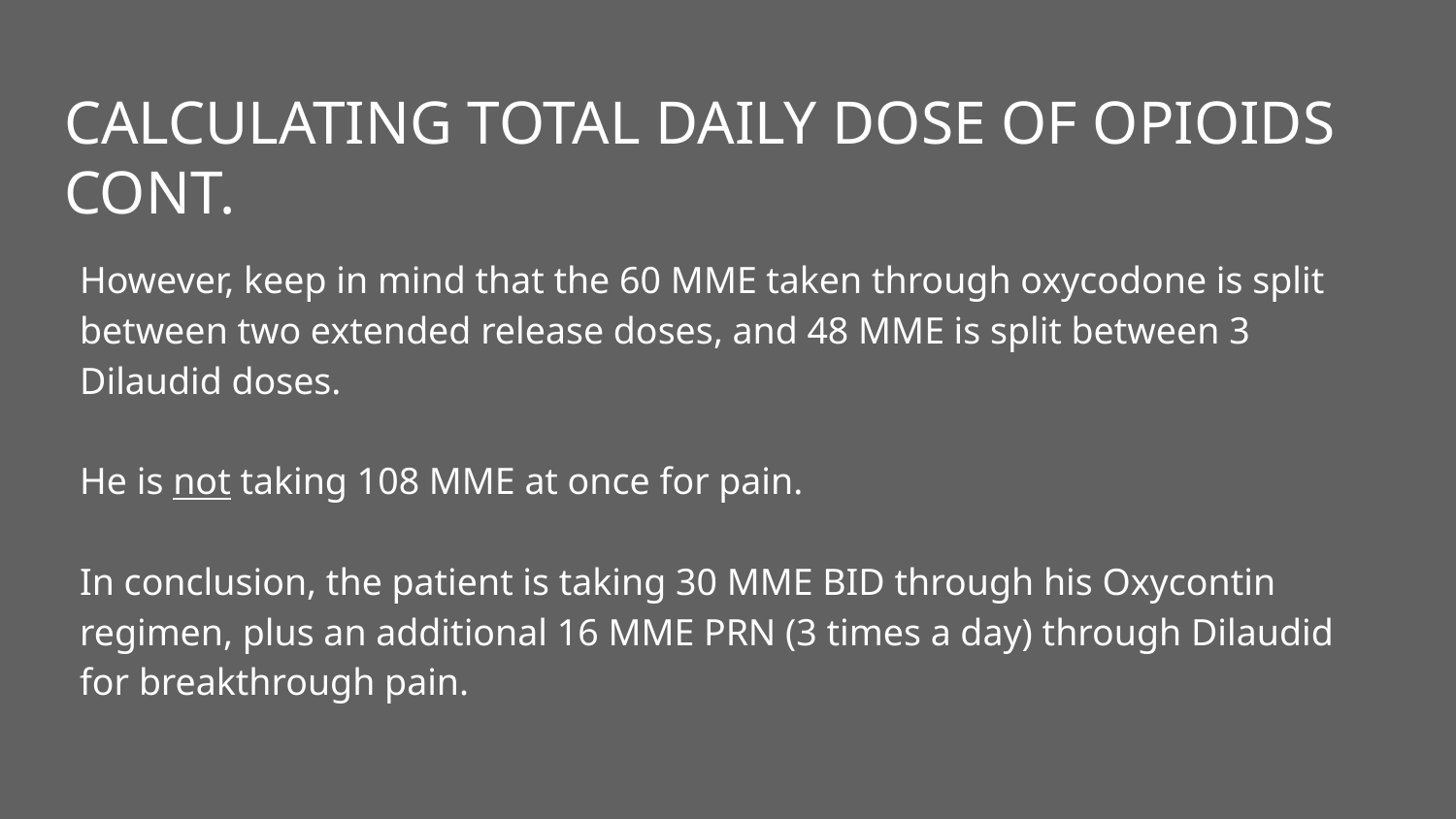

# CALCULATING TOTAL DAILY DOSE OF OPIOIDS CONT.
However, keep in mind that the 60 MME taken through oxycodone is split between two extended release doses, and 48 MME is split between 3 Dilaudid doses.
He is not taking 108 MME at once for pain.
In conclusion, the patient is taking 30 MME BID through his Oxycontin regimen, plus an additional 16 MME PRN (3 times a day) through Dilaudid for breakthrough pain.

## Slide 22
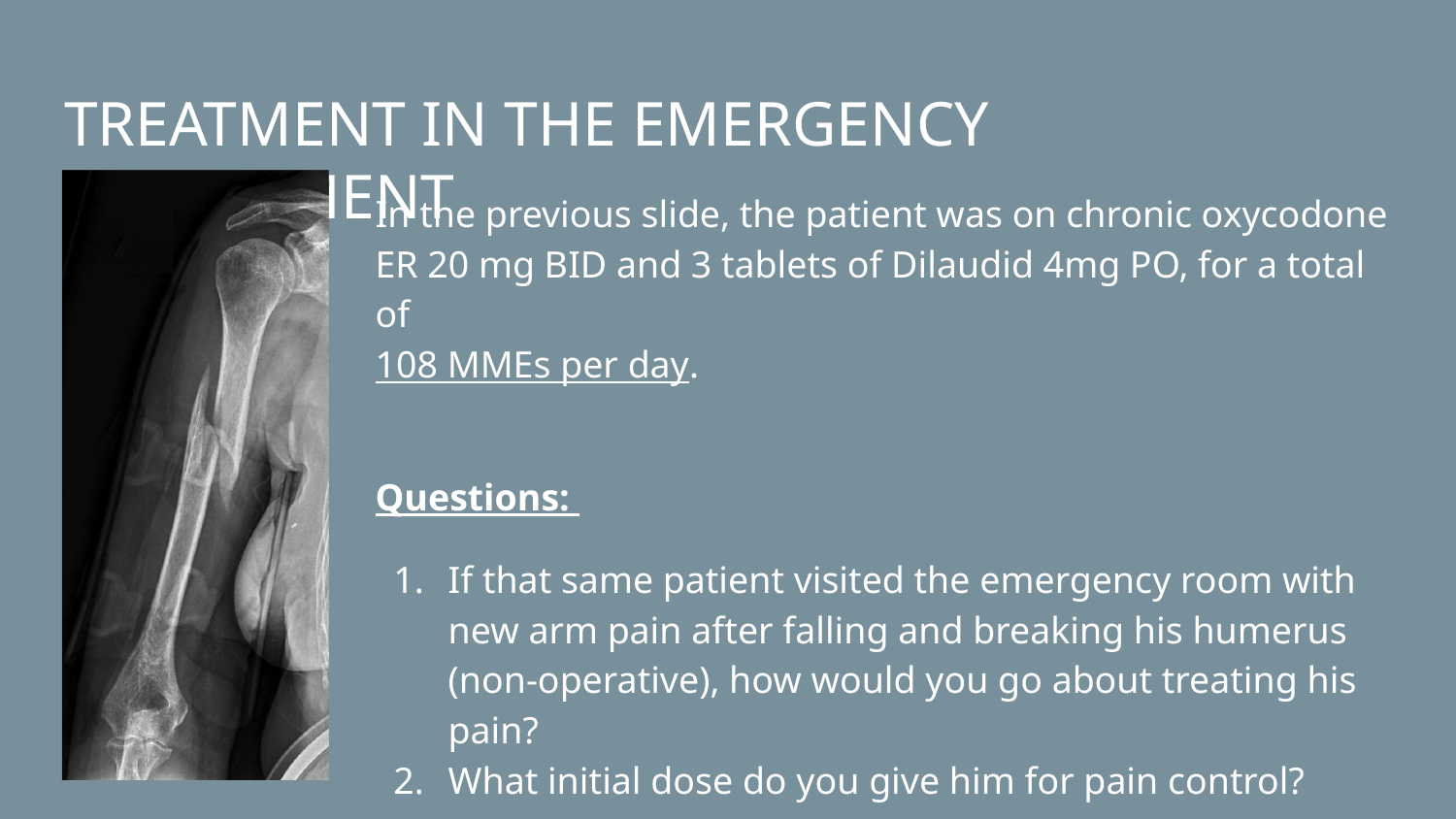

# TREATMENT IN THE EMERGENCY DEPARTMENT
In the previous slide, the patient was on chronic oxycodone ER 20 mg BID and 3 tablets of Dilaudid 4mg PO, for a total of
108 MMEs per day.
Questions:
If that same patient visited the emergency room with new arm pain after falling and breaking his humerus (non-operative), how would you go about treating his pain?
What initial dose do you give him for pain control?

## Slide 23
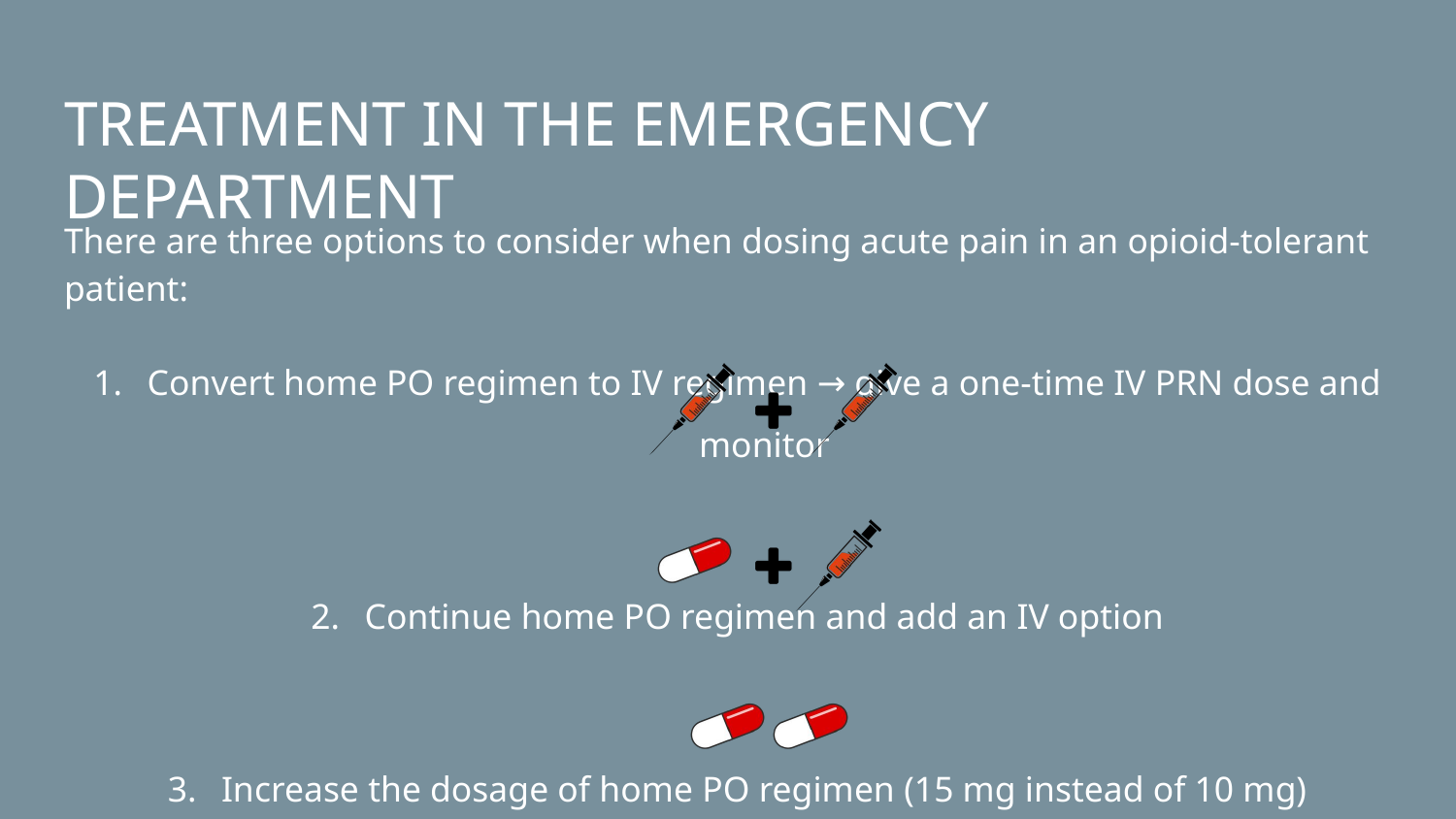

# TREATMENT IN THE EMERGENCY DEPARTMENT
There are three options to consider when dosing acute pain in an opioid-tolerant patient:
Convert home PO regimen to IV regimen → give a one-time IV PRN dose and monitor
Continue home PO regimen and add an IV option
Increase the dosage of home PO regimen (15 mg instead of 10 mg)

## Slide 24
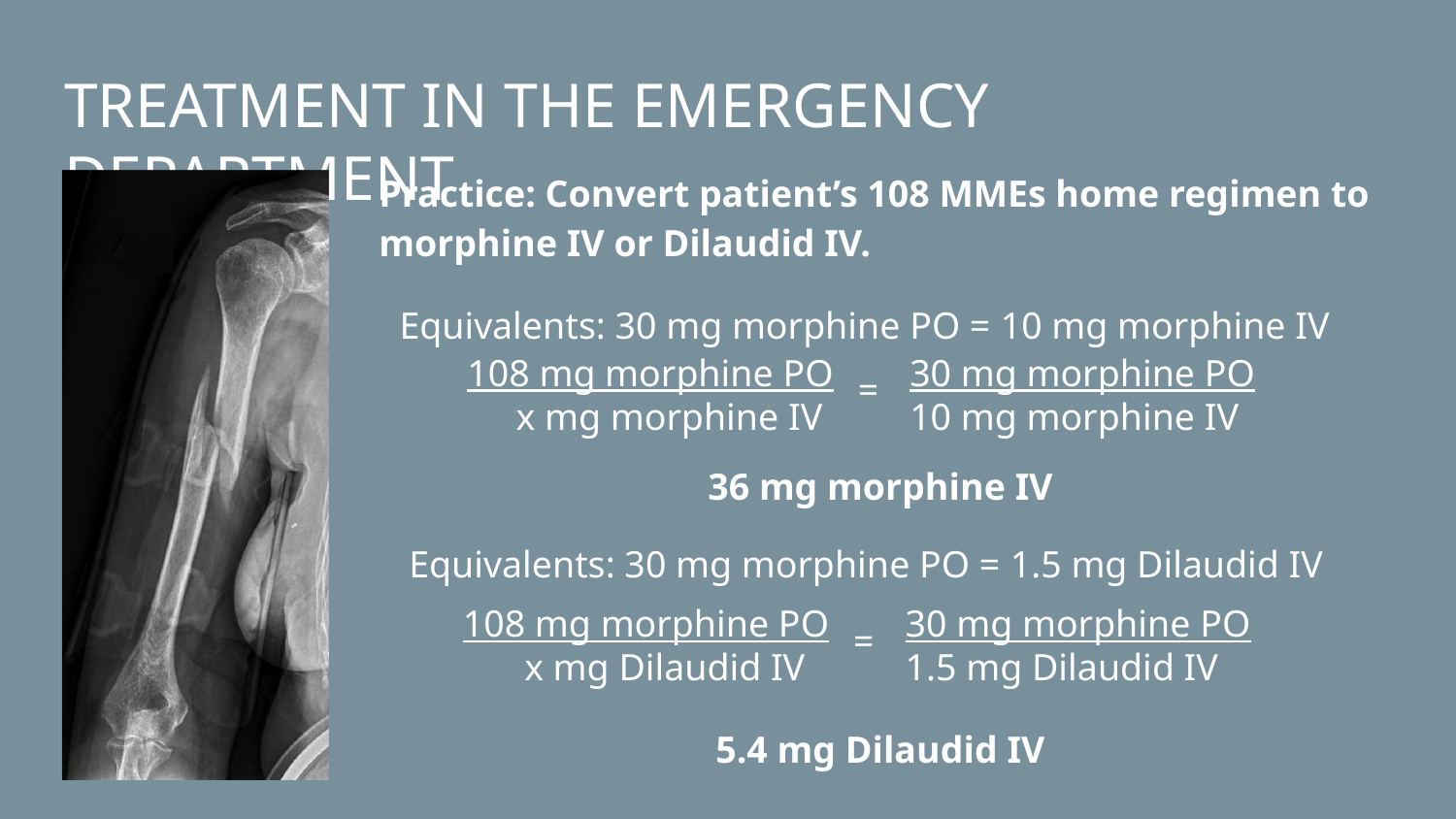

# TREATMENT IN THE EMERGENCY DEPARTMENT
Practice: Convert patient’s 108 MMEs home regimen to morphine IV or Dilaudid IV.
Equivalents: 30 mg morphine PO = 10 mg morphine IV
108 mg morphine PO
 x mg morphine IV
30 mg morphine PO
10 mg morphine IV
=
36 mg morphine IV
Equivalents: 30 mg morphine PO = 1.5 mg Dilaudid IV
108 mg morphine PO
 x mg Dilaudid IV
30 mg morphine PO
1.5 mg Dilaudid IV
=
5.4 mg Dilaudid IV

## Slide 25
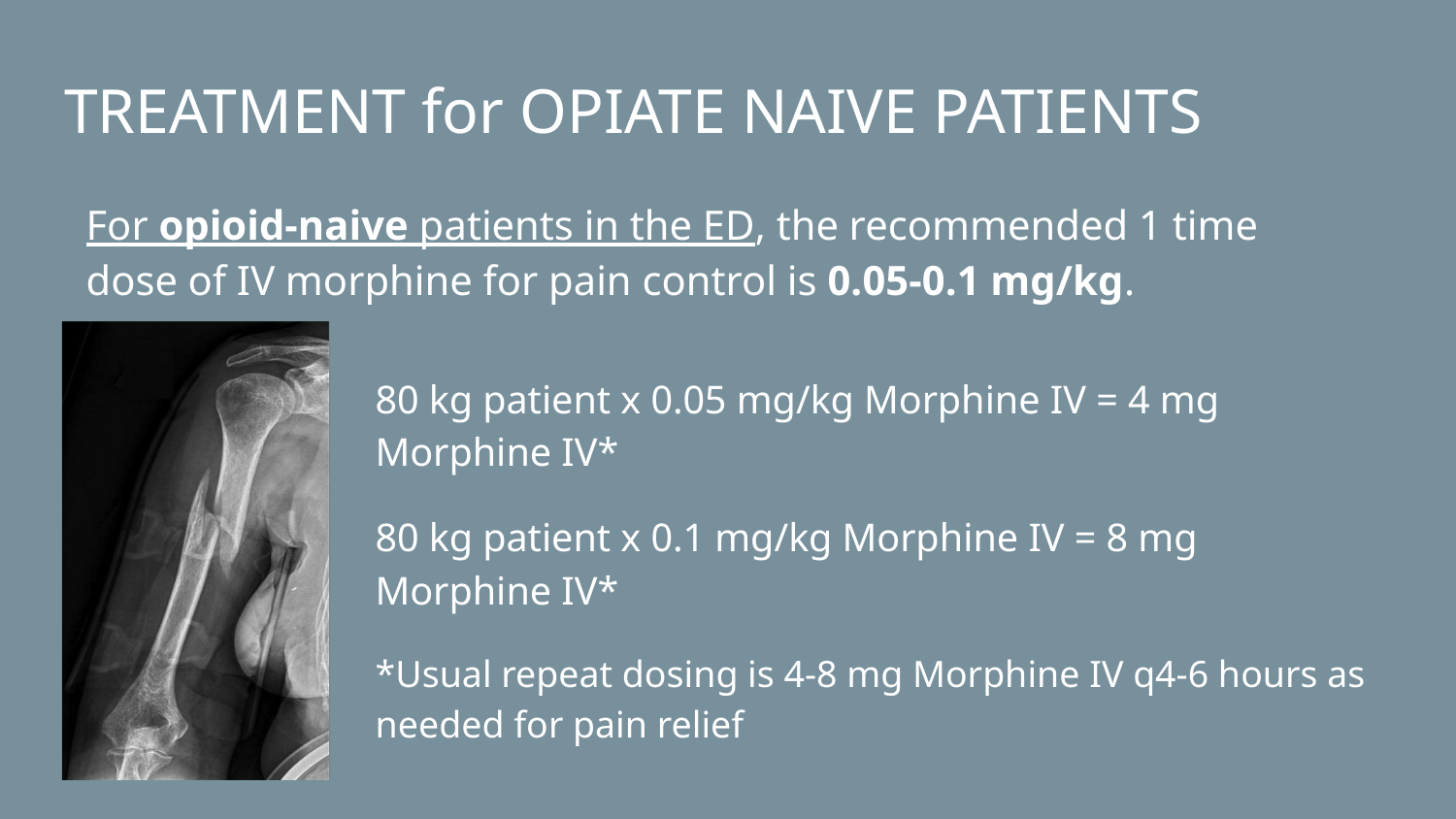

# TREATMENT for OPIATE NAIVE PATIENTS
For opioid-naive patients in the ED, the recommended 1 time dose of IV morphine for pain control is 0.05-0.1 mg/kg.
80 kg patient x 0.05 mg/kg Morphine IV = 4 mg Morphine IV*
80 kg patient x 0.1 mg/kg Morphine IV = 8 mg Morphine IV*
*Usual repeat dosing is 4-8 mg Morphine IV q4-6 hours as needed for pain relief

## Slide 26
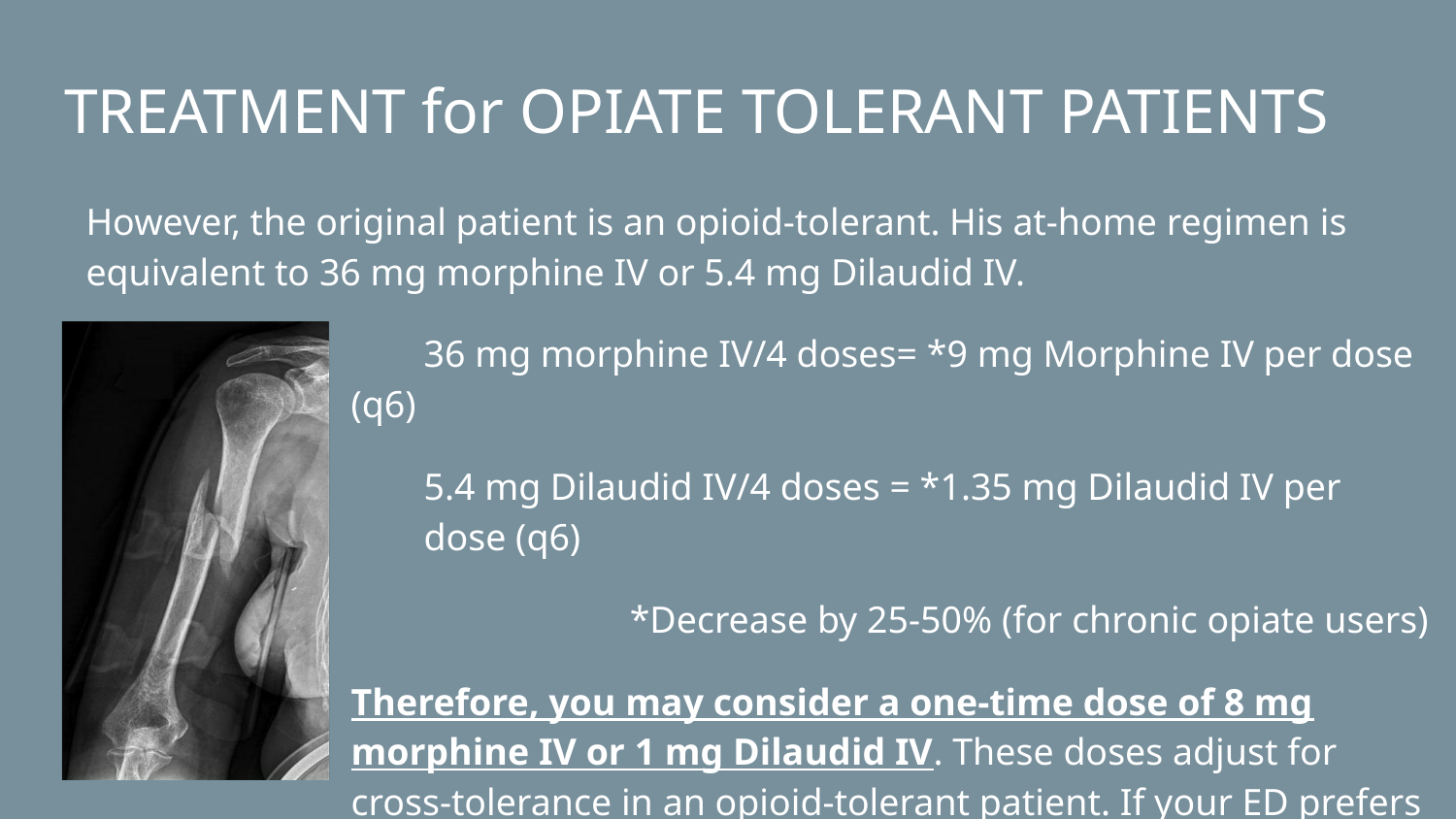

# TREATMENT for OPIATE TOLERANT PATIENTS
However, the original patient is an opioid-tolerant. His at-home regimen is equivalent to 36 mg morphine IV or 5.4 mg Dilaudid IV.
36 mg morphine IV/4 doses= *9 mg Morphine IV per dose (q6)
5.4 mg Dilaudid IV/4 doses = *1.35 mg Dilaudid IV per dose (q6)
*Decrease by 25-50% (for chronic opiate users)
Therefore, you may consider a one-time dose of 8 mg morphine IV or 1 mg Dilaudid IV. These doses adjust for cross-tolerance in an opioid-tolerant patient. If your ED prefers to schedule doses, you can schedule as q4-6 instead of the one-time dose.

## Slide 27
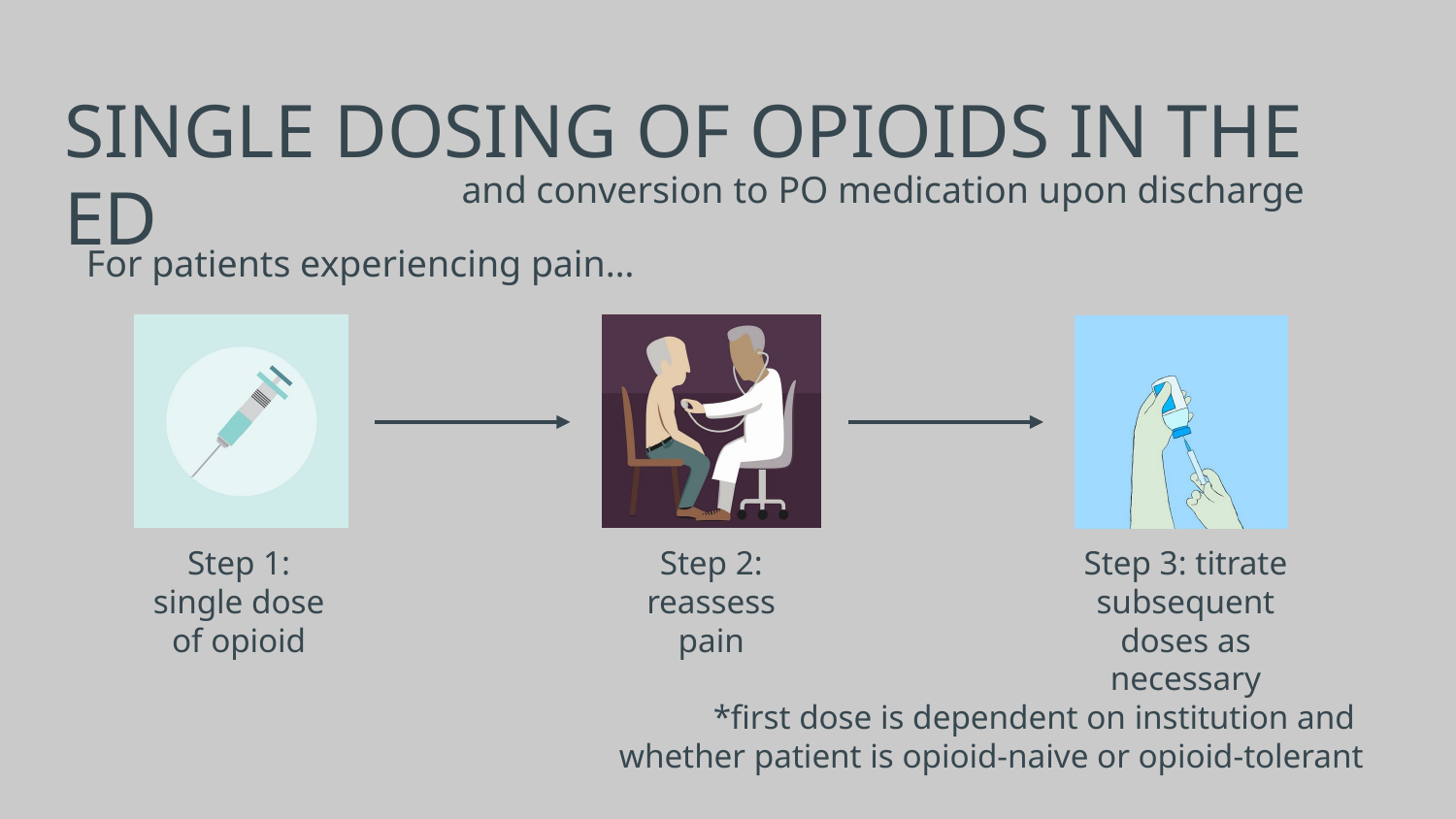

# SINGLE DOSING OF OPIOIDS IN THE ED
and conversion to PO medication upon discharge
For patients experiencing pain…
Step 1: single dose of opioid
Step 2: reassess pain
Step 3: titrate subsequent doses as necessary
*first dose is dependent on institution and
whether patient is opioid-naive or opioid-tolerant

## Slide 28
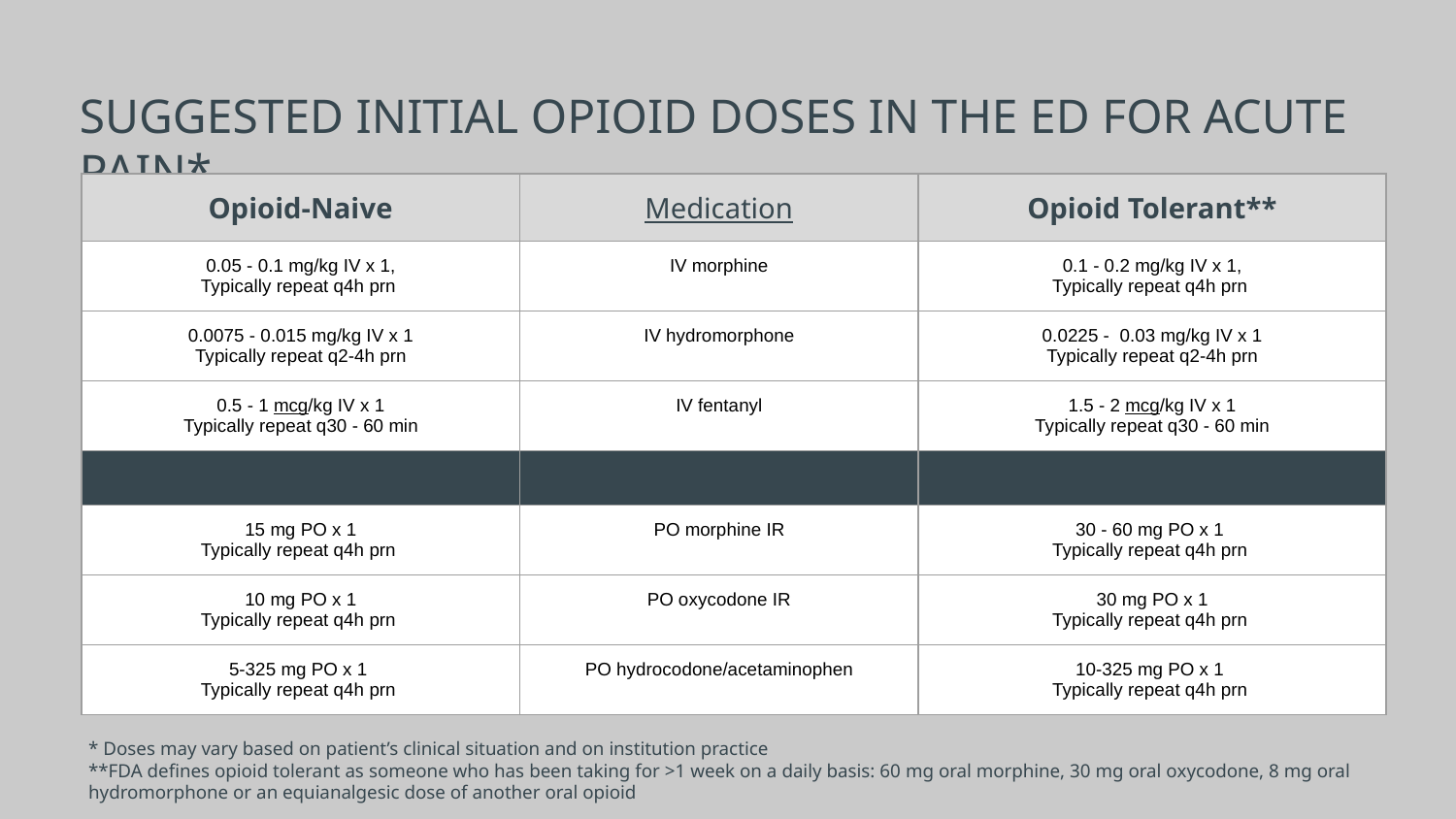

# SUGGESTED INITIAL OPIOID DOSES IN THE ED FOR ACUTE PAIN*
| Opioid-Naive | Medication | Opioid Tolerant\*\* |
| --- | --- | --- |
| 0.05 - 0.1 mg/kg IV x 1, Typically repeat q4h prn | IV morphine | 0.1 - 0.2 mg/kg IV x 1, Typically repeat q4h prn |
| 0.0075 - 0.015 mg/kg IV x 1 Typically repeat q2-4h prn | IV hydromorphone | 0.0225 - 0.03 mg/kg IV x 1 Typically repeat q2-4h prn |
| 0.5 - 1 mcg/kg IV x 1 Typically repeat q30 - 60 min | IV fentanyl | 1.5 - 2 mcg/kg IV x 1 Typically repeat q30 - 60 min |
| | | |
| 15 mg PO x 1 Typically repeat q4h prn | PO morphine IR | 30 - 60 mg PO x 1 Typically repeat q4h prn |
| 10 mg PO x 1 Typically repeat q4h prn | PO oxycodone IR | 30 mg PO x 1 Typically repeat q4h prn |
| 5-325 mg PO x 1 Typically repeat q4h prn | PO hydrocodone/acetaminophen | 10-325 mg PO x 1 Typically repeat q4h prn |
* Doses may vary based on patient’s clinical situation and on institution practice
**FDA defines opioid tolerant as someone who has been taking for >1 week on a daily basis: 60 mg oral morphine, 30 mg oral oxycodone, 8 mg oral hydromorphone or an equianalgesic dose of another oral opioid

## Slide 29
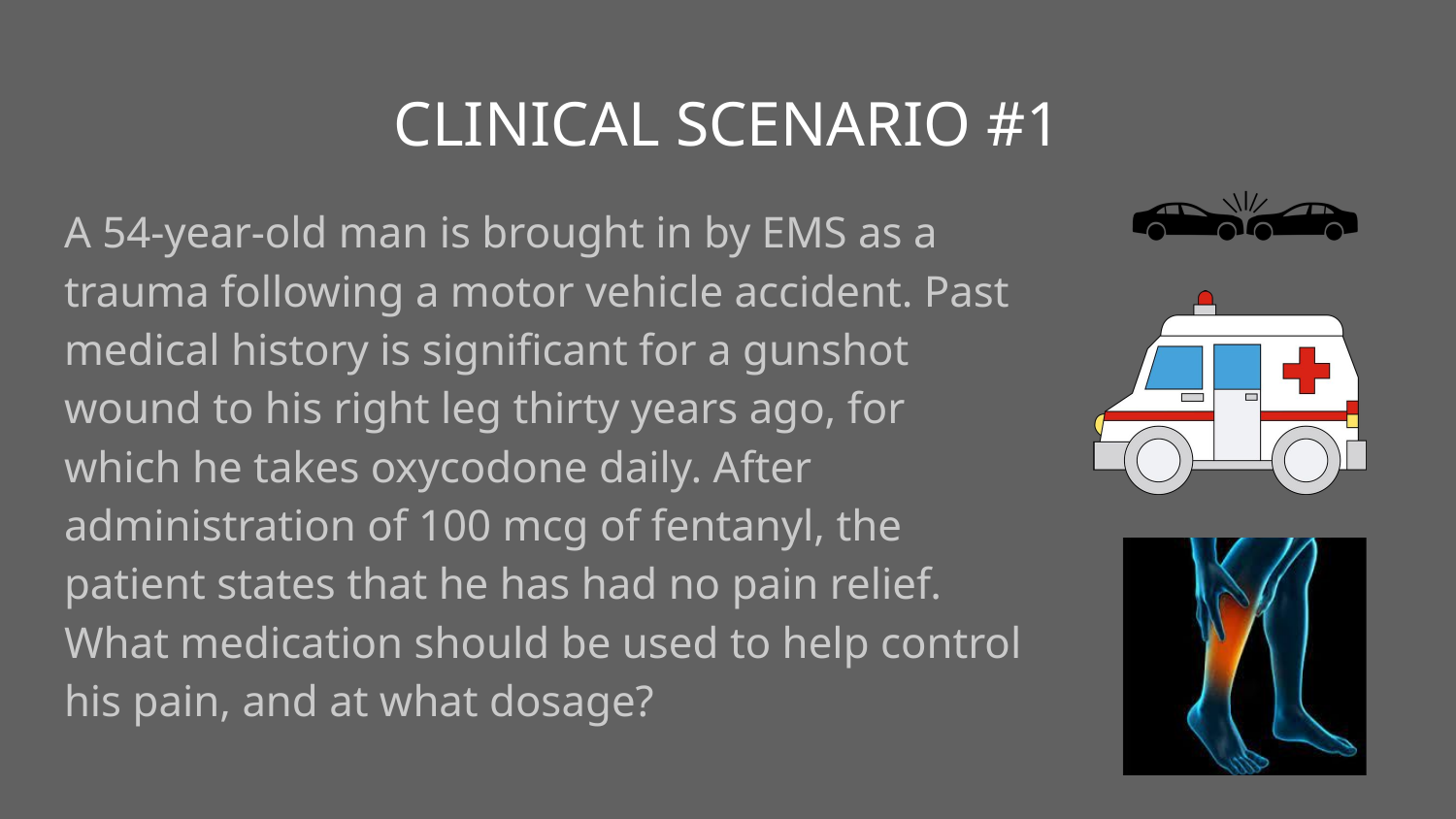

# CLINICAL SCENARIO #1
A 54-year-old man is brought in by EMS as a trauma following a motor vehicle accident. Past medical history is significant for a gunshot wound to his right leg thirty years ago, for which he takes oxycodone daily. After administration of 100 mcg of fentanyl, the patient states that he has had no pain relief. What medication should be used to help control his pain, and at what dosage?

## Slide 30
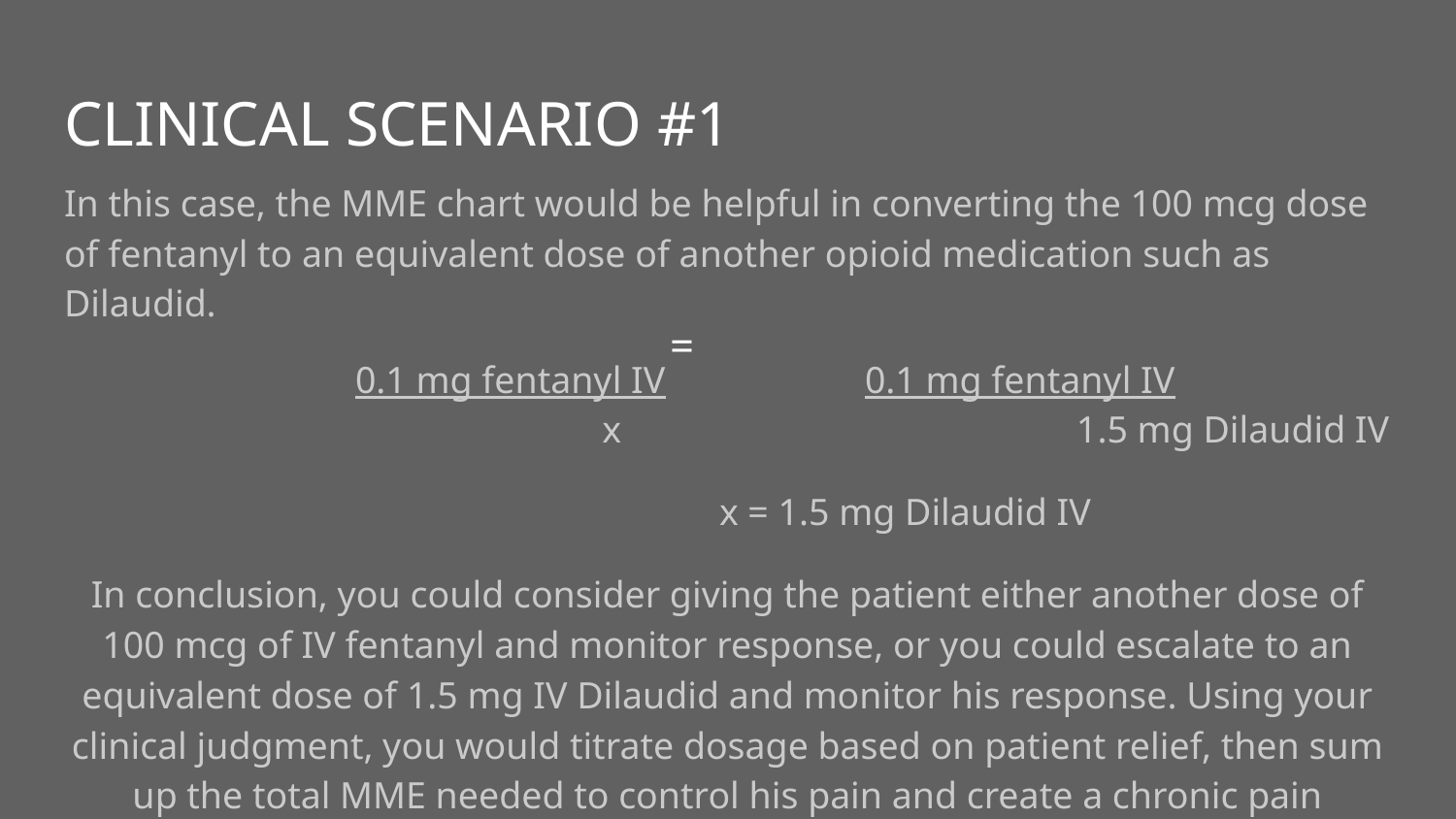

# CLINICAL SCENARIO #1
In this case, the MME chart would be helpful in converting the 100 mcg dose of fentanyl to an equivalent dose of another opioid medication such as Dilaudid.
0.1 mg fentanyl IV		0.1 mg fentanyl IV
			 x			 1.5 mg Dilaudid IV
				x = 1.5 mg Dilaudid IV
In conclusion, you could consider giving the patient either another dose of 100 mcg of IV fentanyl and monitor response, or you could escalate to an equivalent dose of 1.5 mg IV Dilaudid and monitor his response. Using your clinical judgment, you would titrate dosage based on patient relief, then sum up the total MME needed to control his pain and create a chronic pain regimen for the patient upon discharge.
=

## Slide 31
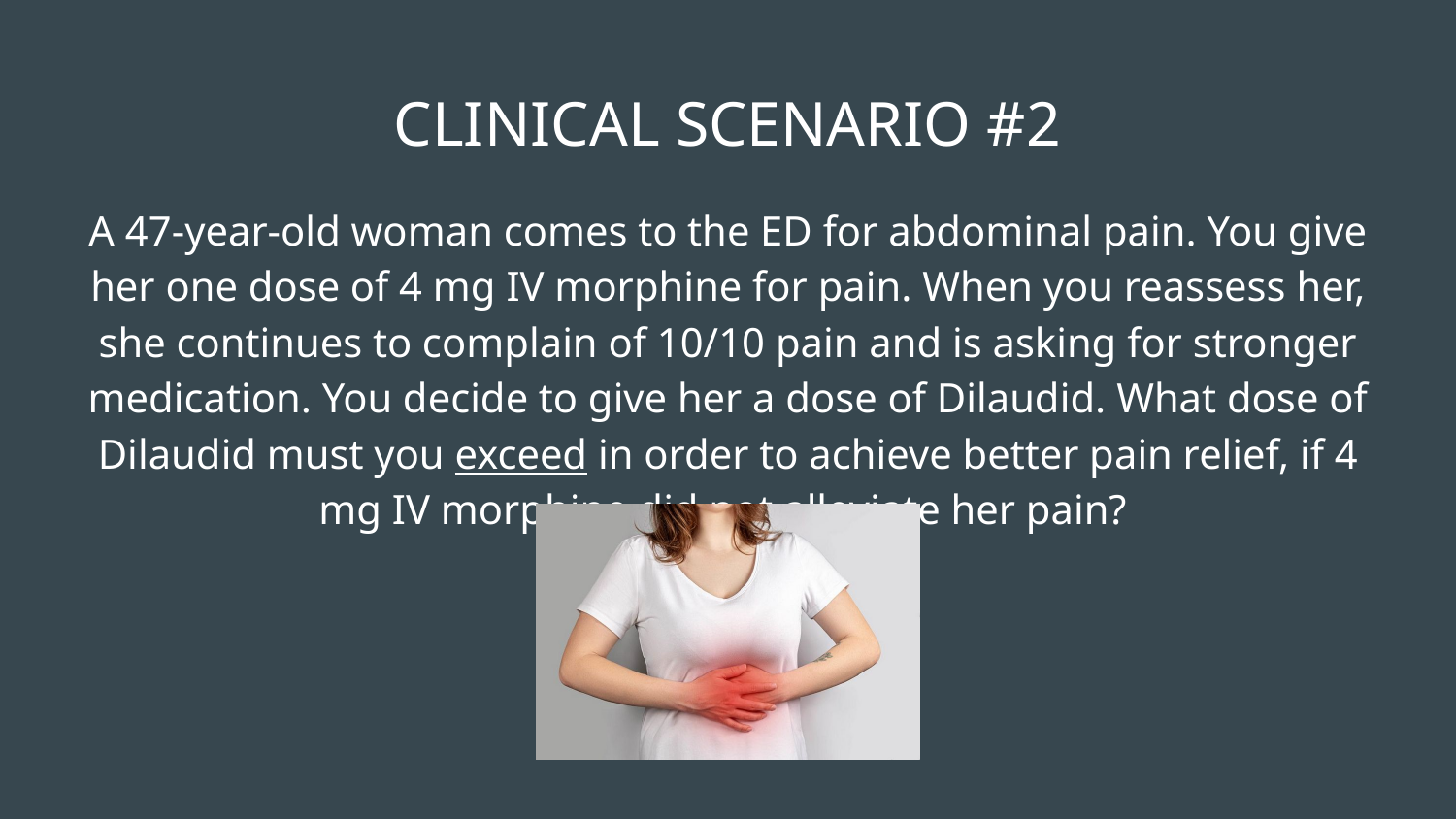

# CLINICAL SCENARIO #2
A 47-year-old woman comes to the ED for abdominal pain. You give her one dose of 4 mg IV morphine for pain. When you reassess her, she continues to complain of 10/10 pain and is asking for stronger medication. You decide to give her a dose of Dilaudid. What dose of Dilaudid must you exceed in order to achieve better pain relief, if 4 mg IV morphine did not alleviate her pain?

## Slide 32
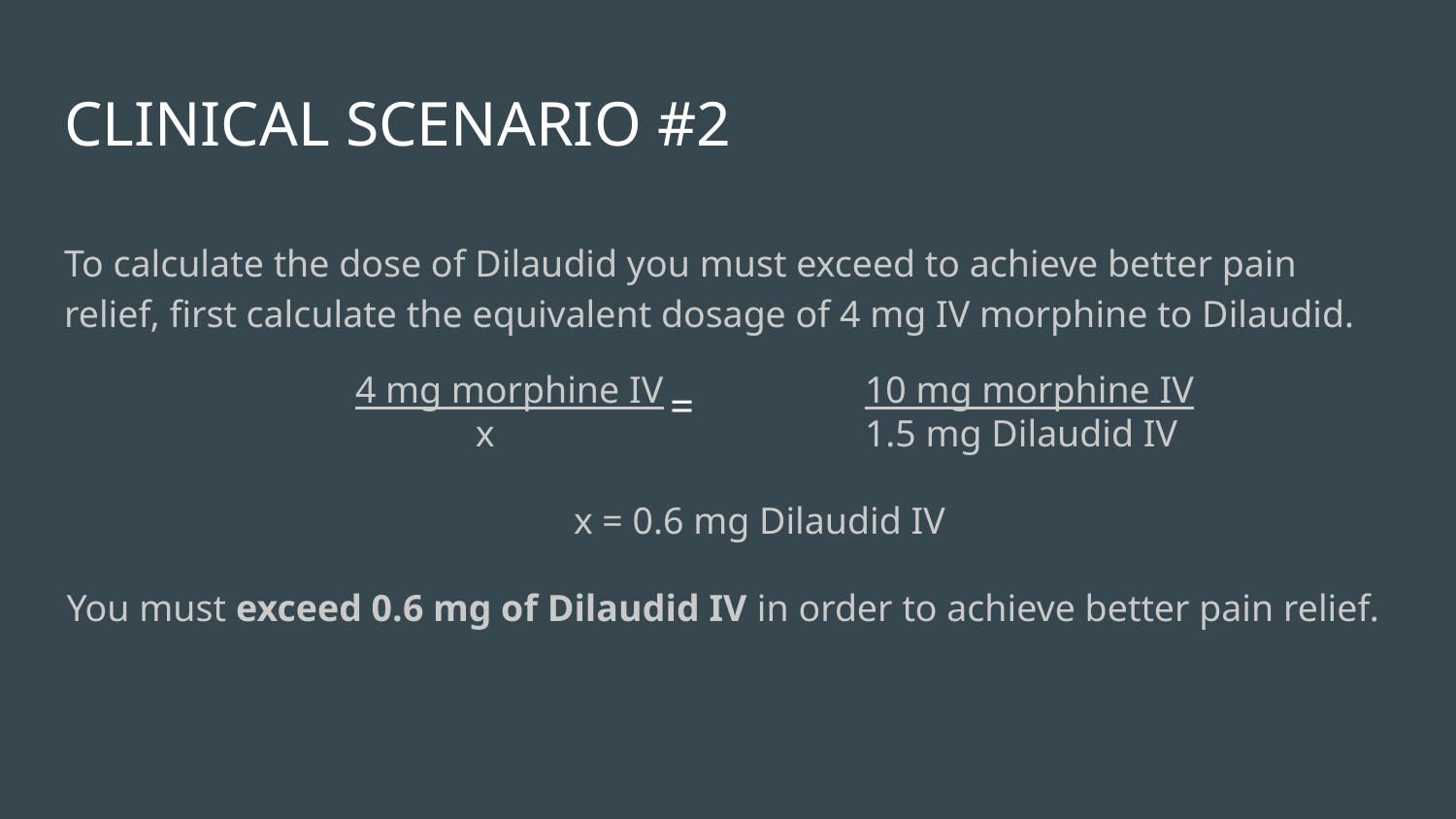

# CLINICAL SCENARIO #2
To calculate the dose of Dilaudid you must exceed to achieve better pain relief, first calculate the equivalent dosage of 4 mg IV morphine to Dilaudid.
4 mg morphine IV		10 mg morphine IV
	 x 			1.5 mg Dilaudid IV
		x = 0.6 mg Dilaudid IV
You must exceed 0.6 mg of Dilaudid IV in order to achieve better pain relief.
=

## Slide 33
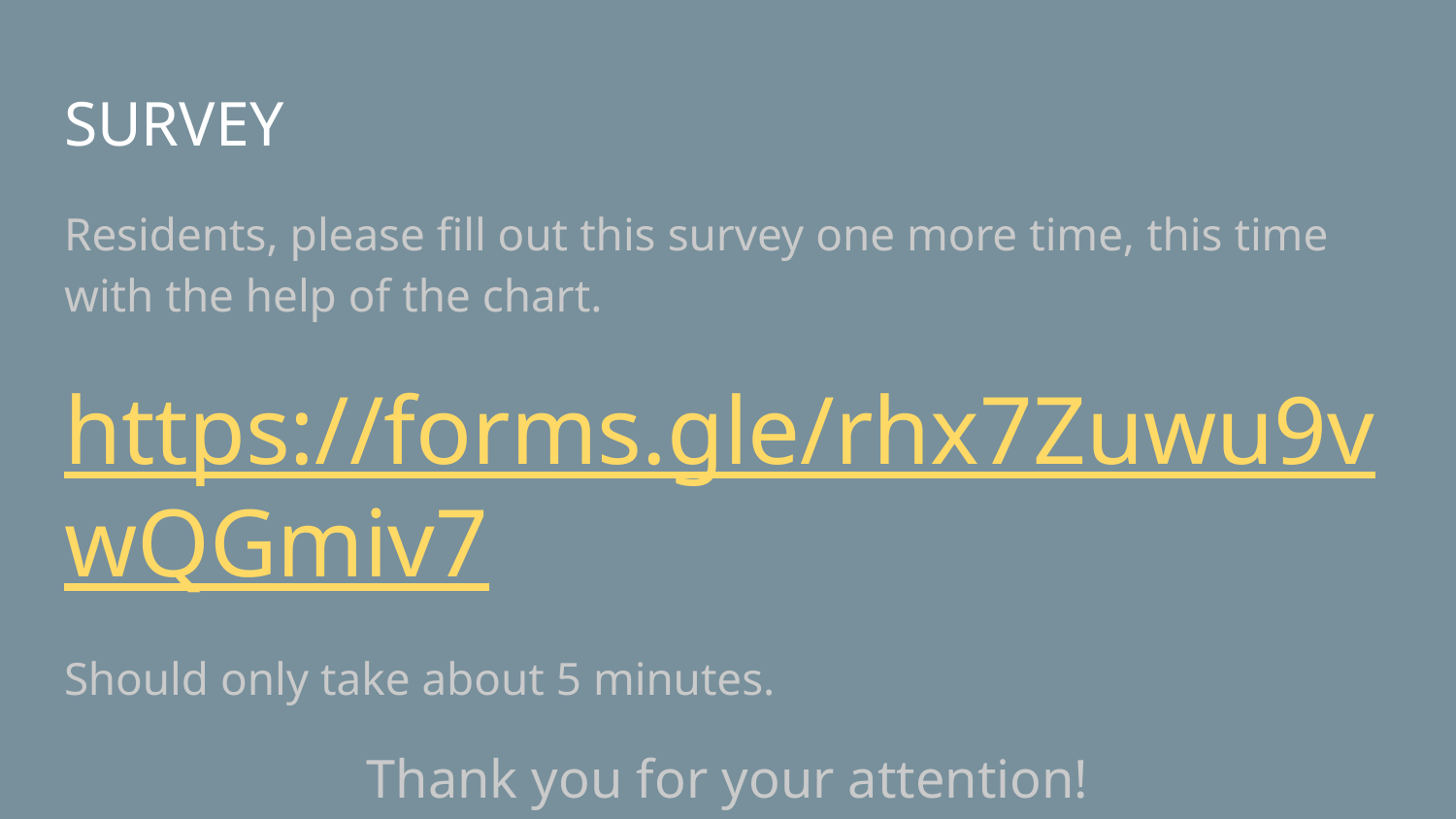

# SURVEY
Residents, please fill out this survey one more time, this time with the help of the chart.
https://forms.gle/rhx7Zuwu9vwQGmiv7
Should only take about 5 minutes.
Thank you for your attention!
