# Supplementary figures and images for: Morphine Equianalgesic Dose Chart in the Emergency Department
Source: J Educ Teach Emerg Med. 2022 Jul 15;7(3):L1–L20. doi: 10.21980/J8RD29 (PMC10332699; doi:10.21980/J8RD29)

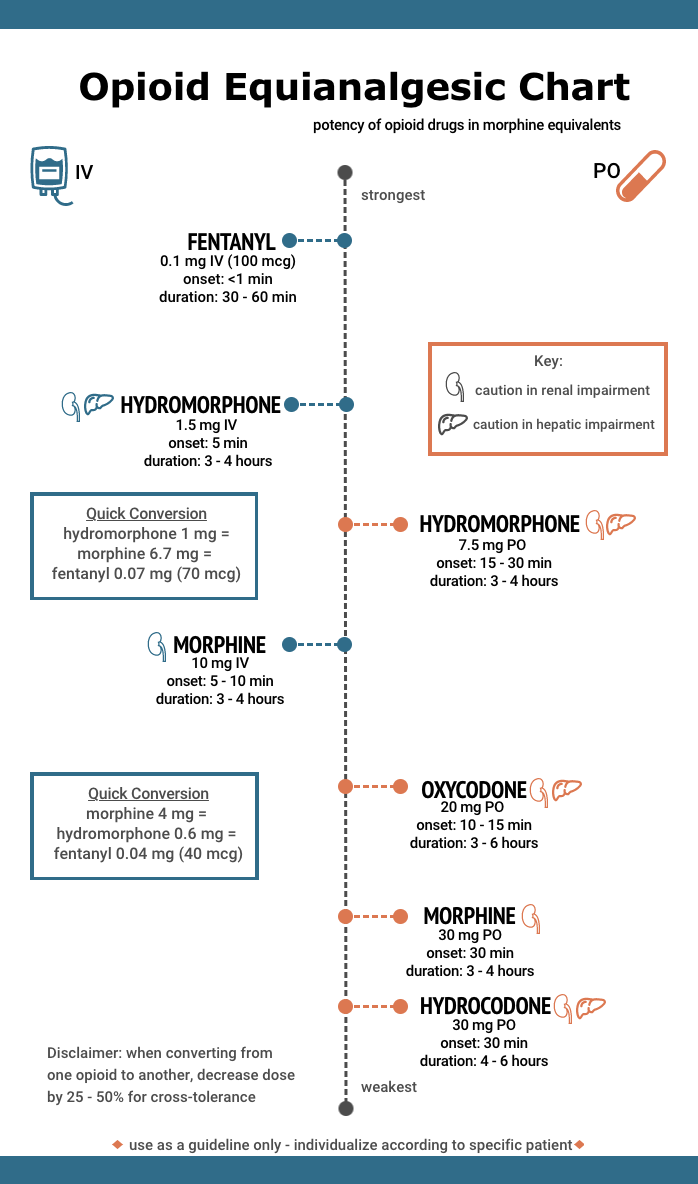

Supplement: Supplementary file 2 — Please see associated PDF file [file jetem-7-3-l1-appendix3.png]
