# Supplementary material for: Morphine Equianalgesic Dose Chart in the Emergency Department
Source: J Educ Teach Emerg Med. 2022 Jul 15;7(3):L1–L20. doi: 10.21980/J8RD29 (PMC10332699; doi:10.21980/J8RD29)
Supplement: Supplementary file 3 — Please see associated PDF file or visit https://www.cdc.gov/opioids/providers/prescribing/guideline.html [file jetem-7-3-l1-appendix4.pdf]

CALCULATING TOTAL DAILY DOSE OF OPIOIDS FOR SAFER DOSAGE

Higher Dosage, Higher Risk.

Higher dosages of opioids are associated with higher risk of overdose and death—even relatively low dosages (20-50 morphine milligram equivalents (MME) per day) increase risk. Higher dosages haven't been shown to reduce pain over the long term. One randomized trial found no difference in pain or function between a more liberal opioid dose escalation strategy (with average final dosage 52 MME) and maintenance of current dosage (average final dosage 40 MME).

Dosages at or **above 50 MME/day** increase risks for overdose by at least

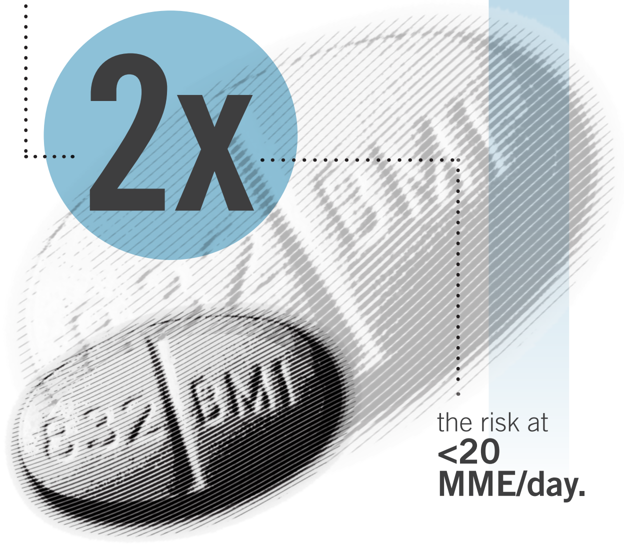

the risk at **<20 MME/day.**

WHY IS IT IMPORTANT TO CALCULATE THE TOTAL DAILY DOSAGE OF OPIOIDS?

Patients prescribed higher opioid dosages are at higher risk of overdose death.

In a national sample of Veterans Health Administration (VHA) patients with chronic pain receiving opioids from 2004–2009, **patients who died** of opioid overdose were prescribed an average of **98 MME/day**, while **other patients** were prescribed an average of **48 MME/day**.

Calculating the total daily dose of opioids helps identify patients who may benefit from closer monitoring, reduction or tapering of opioids, prescribing of naloxone, or other measures to reduce risk of overdose.

HOW MUCH IS 50 OR 90 MME/DAY FOR COMMONLY PRESCRIBED OPIOIDS?

50 MME/day:

- 50 mg of hydrocodone (10 tablets of hydrocodone/acetaminophen 5/300)
- 33 mg of oxycodone (~2 tablets of oxycodone sustained-release 15 mg)
- 12 mg of methadone (<3 tablets of methadone 5 mg)

90 MME/day:

- 90 mg of hydrocodone (9 tablets of hydrocodone/acetaminophen 10/325)
- 60 mg of oxycodone (~2 tablets of oxycodone sustained-release 30 mg)
- ~20 mg of methadone (4 tablets of methadone 5 mg)

U.S. Department of Health and Human Services
Centers for Disease Control and Prevention

LEARN MORE | www.cdc.gov/drugoverdose/prescribing/guideline.html

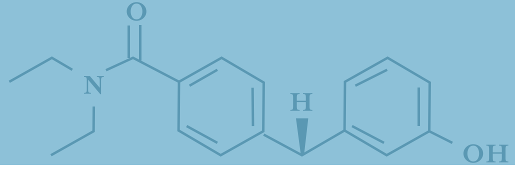

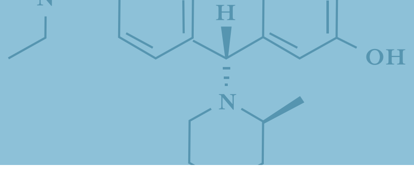

HOW SHOULD THE TOTAL DAILY DOSE OF OPIOIDS BE CALCULATED?

1. **DETERMINE** the total daily amount of each opioid the patient takes.
2. **CONVERT** each to MMEs—multiply the dose for each opioid by the conversion factor. (*see table*)
3. **ADD** them together.

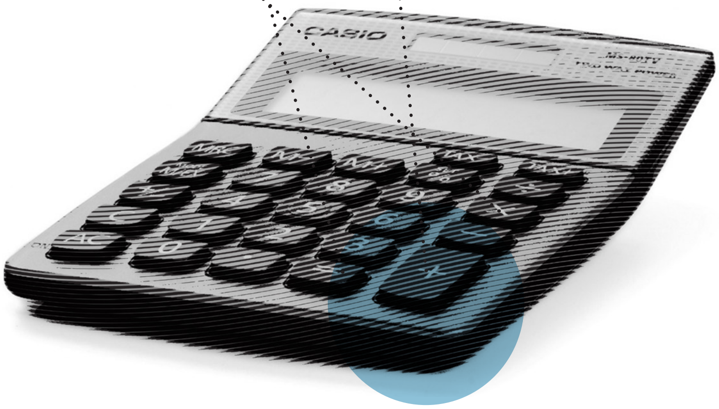

Calculating morphine milligram equivalents (MME)

OPIOID (doses in mg/day except where noted)	CONVERSION FACTOR
Codeine	0.15
Fentanyl transdermal (in mcg/hr)	2.4
Hydrocodone	1
Hydromorphone	4
Methadone	
1-20 mg/day	4
21-40 mg/day	8
41-60 mg/day	10
≥ 61-80 mg/day	12
Morphine	1
Oxycodone	1.5
Oxymorphone	3

These dose conversions are estimated and cannot account for all individual differences in genetics and pharmacokinetics.

CAUTION:

- Do not use the calculated dose in MMEs to determine dosage for converting one opioid to another—the new opioid should be lower to avoid unintentional overdose caused by incomplete cross-tolerance and individual differences in opioid pharmacokinetics. Consult the medication label.

USE EXTRA CAUTION:

- **Methadone:** the conversion factor increases at higher doses
- **Fentanyl:** dosed in mcg/hr instead of mg/day, and absorption is affected by heat and other factors

HOW SHOULD PROVIDERS USE THE TOTAL DAILY OPIOID DOSE IN CLINICAL PRACTICE?

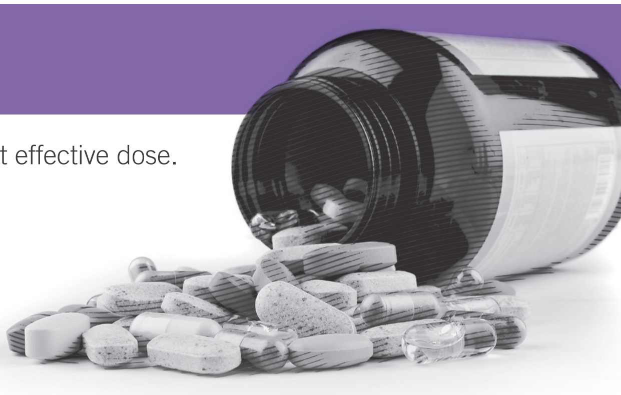

- Use caution when prescribing opioids at any dosage and prescribe the lowest effective dose.
- Use extra precautions when increasing to ≥50 MME per day* such as:
  - Monitor and assess pain and function more frequently.
  - Discuss reducing dose or tapering and discontinuing opioids if benefits do not outweigh harms.
  - Consider offering naloxone.
- Avoid or carefully justify increasing dosage to ≥90 MME/day.*

* These dosage thresholds are based on overdose risk when opioids are prescribed for pain and should not guide dosing of medication-assisted treatment for opioid use disorder.

LEARN MORE | www.cdc.gov/drugoverdose/prescribing/guideline.html
